# Coronavirus disease 2019 (COVID-19) excess mortality outcomes associated with pandemic effects study (COPES): A systematic review and meta-analysis

**DOI:** 10.3389/fmed.2022.999225

**Published:** 2022-12-16

**Authors:** David Lu, Sumeet Dhanoa, Harleen Cheema, Kimberley Lewis, Patrick Geeraert, Benjamin Merrick, Aaron Vander Leek, Meghan Sebastianski, Brittany Kula, Dipayan Chaudhuri, John Basmaji, Arnav Agrawal, Dan Niven, Kirsten Fiest, Henry T. Stelfox, Danny J. Zuege, Oleksa G. Rewa, Sean M. Bagshaw, Vincent I. Lau

**Affiliations:** ^1^Faculty of Medicine and Dentistry, Alberta Health Services, University of Alberta, Edmonton, AB, Canada; ^2^Department of Health Research Methods, Evidence and Impact, McMaster University, Hamilton, ON, Canada; ^3^Division of Critical Care Medicine, Department of Medicine, McMaster University, Hamilton, ON, Canada; ^4^Alberta Strategy for Patient-Oriented Research Knowledge Translation Platform, University of Alberta, Edmonton, AB, Canada; ^5^Division of Infectious Disease, Department of Medicine, Faculty of Medicine and Dentistry, Alberta Health Services, University of Alberta, Edmonton, AB, Canada; ^6^Division of Critical Care, Department of Medicine, Western University, London, ON, Canada; ^7^Division of General Internal Medicine, Department of Medicine, McMaster University, Hamilton, ON, Canada; ^8^Department of Critical Care Medicine, Cumming School of Medicine, Alberta Health Services, University of Calgary, Calgary, AB, Canada; ^9^Department of Critical Care Medicine, Faculty of Medicine and Dentistry, Alberta Health Services, University of Alberta, Edmonton, AB, Canada; ^10^School of Public Health, University of Alberta, Edmonton, AB, Canada

**Keywords:** excess mortality, COVID-19, non-COVID-19 mortality, pandemic (COVID-19), outcomes

## Abstract

**Background and aim:**

With the Coronavirus Disease 2019 (COVID-19) pandemic continuing to impact healthcare systems around the world, healthcare providers are attempting to balance resources devoted to COVID-19 patients while minimizing excess mortality overall (both COVID-19 and non-COVID-19 patients). To this end, we conducted a systematic review (SR) to describe the effect of the COVID-19 pandemic on all-cause excess mortality (COVID-19 and non-COVID-19) during the pandemic timeframe compared to non-pandemic times.

**Methods:**

We searched EMBASE, Cochrane Database of SRs, MEDLINE, Cumulative Index to Nursing and Allied Health Literature (CINAHL) and Cochrane Controlled Trials Register (CENTRAL), from inception (1948) to December 31, 2020. We used a two-stage review process to screen/extract data. We assessed risk of bias using Newcastle-Ottawa Scale (NOS). We used Critical Appraisal and Grading of Recommendations Assessment, Development and Evaluation (GRADE) methodology.

**Results:**

Of 11,581 citations, 194 studies met eligibility. Of these studies, 31 had mortality comparisons (*n* = 433,196,345 participants). Compared to pre-pandemic times, during the COVID-19 pandemic, our meta-analysis demonstrated that COVID-19 mortality had an increased risk difference (RD) of 0.06% (95% CI: 0.06–0.06% *p* < 0.00001). All-cause mortality also increased [relative risk (RR): 1.53, 95% confidence interval (CI): 1.38–1.70, *p* < 0.00001] alongside non-COVID-19 mortality (RR: 1.18, 1.07–1.30, *p* < 0.00001). There was “very low” certainty of evidence through GRADE assessment for all outcomes studied, demonstrating the evidence as uncertain.

**Interpretation:**

The COVID-19 pandemic may have caused significant increases in all-cause excess mortality, greater than those accounted for by increases due to COVID-19 mortality alone, although the evidence is uncertain.

**Systematic review registration:**

[https://www.crd.york.ac.uk/prospero/#recordDetails], identifier [CRD42020201256].

## Highlights

– **Question:** What was the total burden of all-cause mortality during the COVID-19 pandemic, inclusive of COVID-19 mortality, non-COVID-19 mortality, and all-cause mortality?
– **Findings:** In this systematic review of 31 observational studies (*n* = 433,196,345 participants), there was a significant increase in all-cause excess mortality through the COVID-19 pandemic as compared to pre-pandemic time periods. This increase in excess mortality is only partially explained by COVID-19 deaths, as there was a substantial number of non-COVID-19 patients affected by the pandemic time period as well.
– **Meaning:** The COVID-19 pandemic may have caused a significant amount of death, both from COVID-19 related illness and non-COVID-19 illness alike. Monitoring all-cause excess mortality may be a better measure to ascertain the COVID-19 pandemic’s full impact on mortality.

## Introduction

As of June 23, 2022, the World Health Organization (WHO) has reported over 545 million cases and over 6.3 million deaths from coronavirus disease 2019 (COVID-19) (1). However, assessing the true mortality from COVID-19 is challenging. Confounding factors include: (1) lack of testing availability or policies leading to undertesting (2); (2) varying COVID-19 diagnostic criteria (e.g., suspected cases vs. confirmed cases, nucleic acid vs. antigen vs. antibody testing) (3); (3) testing sensitivity and specificity (e.g., rapid antigen test vs. polymerase chain reaction) (4–6). This can create uncertainty in producing accurate mortality data surrounding the pandemic. There is evidence to suggest non-COVID-19 mortality was excessive during the COVID-19 pandemic, due to disruptions in healthcare provisions and changes in acute care hospitalizations, leading to increased all-cause morbidity and mortality (7). Therefore, following COVID-19 mortality alone does not accurately estimate the full impact of the pandemic.

It is crucial to evaluate total excess (e.g., avoidable or unanticipated) mortality during the pandemic, as compared to non-pandemic times (8, 9). Multiple studies have demonstrated that COVID-19 deaths can be underestimated (10–12), where measuring total excess mortality can better capture the total impact of the pandemic, and total excess mortality may be a worthwhile measure to calculate attributable mortality from the pandemic (13). The ability to adapt and innovate during these periods of global disruption is key to mitigating the adverse effects of the pandemic on global health, both related to the immediate and prolonged complications of COVID-19, alongside any negative pandemic influences on non-COVID-19 mortality (7).

To this end, we conducted a systematic review (SR) to describe the total burden of all-cause excess mortality during the pandemic (inclusive of both COVID-19 and non-COVID-19 mortality). The purpose of this SR is to inform clinicians, health policymakers (e.g., public health officials, ethicists, politicians), and patients to ensure that policies enacted are representative of the full breadth of impact of the pandemic for all patients.

## Methods

### Population, intervention, comparison, and outcomes (PICO)

In populations around the world [population], what impact did the COVID-19 pandemic (intervention) as compared to the pre-pandemic era (comparator) have on excess mortality of both COVID-19 and non-COVID-19 patients (outcomes) during pre-pandemic and pandemic periods (time)?

### Search and inclusion criteria

This SR was conducted in line with Preferred Reporting Items for Systematic Reviews and Meta-Analyses (PRISMA) guidelines (14), with International Prospective Register of Systematic Reviews (PROSPERO) on September 2, 2020 (CRD42020201256). Our PRISMA checklist is included in Supplementary Table 1. This paper specifically focused on COVID-19 related excess mortality alongside all-cause excess mortality (with non-COVID-19 mortality) (7).

We systematically searched Ovid EMBASE, Cochrane Database of Systematic Reviews, MEDLINE, Cumulative Index to Nursing and Allied Health Literature (CINAHL) and Cochrane Controlled Trials Register (CENTRAL), from inception (1948) to December 31, 2020. Searches were performed by a clinical librarian (DKL) and underwent Peer Review Electronic Search Strategy (PRESS) (15) by a second researcher (MS). The search criteria are summarized in Supplementary Appendix 1.

The following keywords (topic/subject and keywords) were used, alongside alternative word spellings and endings: *excess mortality*; *pandemic*; *non-pandemic time periods*; *outcomes*. For each specific electronic database, individual search parameters were adjusted for syntax, field names, and search terms. Supplementary searches and bibliographies of relevant studies were also explored.

### Operational definitions

The COVID-19 pandemic exposure time period was defined as December 31, 2019 (first initial reports of pneumonia of unknown etiology to the WHO) (16), and forward. The non-COVID-19 pandemic control time period was defined as before December 31, 2019 (7). There was variability among the time periods cited, hence a broader definition was chosen to include studies that used data early in the pandemic.

### Eligibility criteria

Inclusion criteria were: (1) randomized control trials (RCTs), (2) observational studies and case series with control groups; (3) adult patients (>18 years old); and (4) must compare mortality outcomes in time periods during and before the COVID-19 pandemic. All animal and pediatric studies were excluded. We excluded all non-peer reviewed websites and non-research conference abstracts. No language restrictions were applied.

### Study selection and data abstraction

At least two independent reviewers (DL, SD, HC, PG, BM, AV, KL, BK, DC, and AA) evaluated and assessed for eligibility of each of the citations and utilized the previous eligibility criteria. Any citations selected by either reviewer was advanced to the full-text screening second stage. In the second stage, at least two reviewers reviewed the full-text articles for inclusion. Discussion with a third reviewer (VL) was used to resolve conflicts, if necessary. To manage screening and selection of studies, Covidence (Veritas Health Innovation, Melbourne, VIC, Australia) was used (17).

We developed an *a priori* data abstraction tool, which was piloted among all data abstractors (most demographics and baseline characteristics were extractable, however, excess mortality data were not routinely reported for pandemic and pre-pandemic time periods, limiting the number of studies pooled for analysis). We abstracted the following data points from included articles: study characteristics (title, author), patient group demographic/clinical data, interventions and comparators, clinical outcome data: mortality and jurisdiction(s) in which the study was performed. Supplementary Tables 2, 3 outline our data abstraction.

### Risk of bias assessment

We used the Newcastle-Ottawa Scale (NOS) to evaluate the risk of bias (RoB) in non-randomized observational case-control and cohort studies. The following domains were assessed: selection (maximum score: four), comparability (maximum: two), and exposure (maximum: three). Each NOS scales for case-control/cohort studies are outlined in the footnotes of Supplementary Tables 4A, B (18). Study quality was deemed either: poor (selection domain: 0–1 star, comparability: 0, exposure: 0–1); fair (selection: 2, comparability: 1–2, exposure: 2–3); or good (selection: 3–4 domain, comparability: 1–2, exposure: 2–3) (18).

### Grading of recommendations assessment, development, and evaluation

Grading of Recommendations Assessment, Development, and Evaluation (GRADE) methodology was used to assess publication bias, inconsistency, imprecision, indirectness, and RoB for the clinical outcomes of all-cause mortality, COVID-19 mortality, and non-COVID-19 mortality. Certainty of evidence was rated as high, moderate, low, or very low, with RCTs initially rated as high, and observational studies initially rated as low (19–21).

### Data synthesis and analysis

Continuous variables are displayed as medians and inter-quartile ranges (IQR) and/or means and standard deviations (SD), where appropriate. Comparisons were performed using a Wilcoxon rank sum test. Categorical variables were presented as counts and proportions and were assessed using Fischer’s exact tests or Pearson’s chi-squared test, where appropriate.

RevMan version 5.4 software (Copenhagen: The Nordic Cochrane Centre, Cochrane Collaboration 2014) was used to perform meta-analysis. The DerSimonian and Laird methodology was used to pool effect sizes. Study weights were measured using the inverse variance method using a random-effects model (22). Results were presented as relative risk (RR) or mean difference with 95% confidence intervals (CIs) (23). Heterogeneity was evaluated using the *I*^2^ statistic (>50% demonstrating significant heterogeneity). We also assessed the χ2 test for homogeneity (*p* < 0.1 demonstrating substantial heterogeneity). Subgroup analyses were attempted to determine if there were any methodological or clinical sources of heterogeneity. Publication bias was not formally assessed because in the meta-analysis there were fewer than 10 studies per outcome (23–25). However, we do present the funnel plots in Supplementary Figures 2A–C.

**FIGURE 1:**
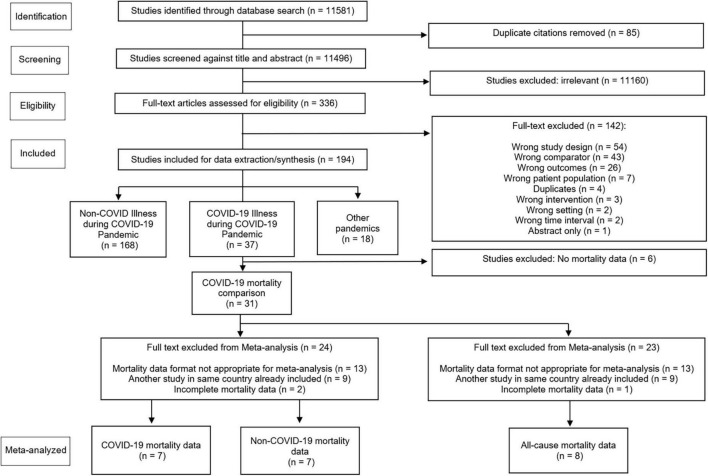
COPES PRISMA flow diagram.

Due to the variations in reporting among the included studies, meta-analysis was completed for a subset of studies. The reasons for exclusion are outlined in Figure 1, which included inappropriate data format (did not include COVID-19 death, non-COVID-19 deaths, or all-cause deaths), duplication of data sets such as overlapping jurisdictions (only included the study with the largest number of participants), and incomplete data. We narratively summarized the eligible studies in terms of point estimates or proportions with *p*-values or 95% CIs. Significance was set at 0.05.

### Subgroup analyses

Potential methodologic or clinical sources of heterogeneity were examined to determine if significant heterogeneity existed. If enough trials were available, we attempted pre-specified subgroup analyses (hypothesized direction in parentheses) were considered for comparison:

• Low-middle income countries (LMIC) vs. high-income countries (HIC), as per the World Bank definition (26) (outcomes would favor high-income countries during both pandemic and non-pandemic times).
• High vs. low RoB studies (high RoB studies would favor non-pandemic usual care management outcomes).
• Medical vs. surgical vs. medical/surgical case-mixes (surgical cases would be favored during pandemic times compared to medical cases).
• Randomized control trials vs. observational studies (observational studies would favor non-pandemic usual care mortality outcomes).
• Pandemic time periods vs. non-pandemic time periods (would favor non-pandemic time period outcomes).

If a subgroup’s effects were deemed to be credible, outcomes were presented separately for each subgroup.

### Dealing with missing data

We attempted to contact the study authors for any missing data. If unavailable, we presented what data is available, and commented in the discussion on the potential impact of the missing data.

## Results

### Study characteristics

We identified 11,581 articles during our database search. Of these, 336 full texts were reviewed, with 31 eligible studies reporting mortality data (Figure 1). Table 1 presents a summary of the study characteristics. Supplementary Tables 2, 3 show complete study data, demographics, baseline characteristics, subgroups, and outcomes.

**TABLE 1:** Summary statistics of study design and characteristics of the studies included in COPES (*n* = 31).

Publication status	*n* (%)	Setting	
Peer-reviewed publication	30 (96.4%)	Acute care hospital	13 (41.9%)
Pre-print	1 (3.6%)	Emergency department	1 (3.2%)
		Ward	0 (0.0%)
Study design		Intensive care unit	2 (6.5%)
Observational (cohort)	30 (97.6%)	Other/not applicable	15 (48.4%)
Observational (case-control)	1 (1.2%)		
		Country	*n* (%)
REB approval		Multinational	3 (9.7%)
Yes	6 (19.4%)	Single	28 (90.3%)
Not required	14 (45.2%)		
Not reported	9 (29.0%)	Subgroups:	
Not applicable	2 (6.5%)	Risk of bias	
		Good	3 (9.7%)
Consent obtained		Fair	0
Yes	1 (3.3%)	Poor	28 (90.3%)
No	14 (45.2%)		
Not reported	8 (25.8%)	High vs. low/middle-income country	
Not applicable	8 (25.8%)	High	27 (87.1%)
		Low/middle	4 (12.9%)
**Funding**			
Industry	0 (0.0%)	Case-mix	
Government	9 (29.0%)	Medical	7 (22.6%)
Institutional	5 (16.1%)	Surgical	0
Non-for-profit	3 (9.7%)	Mixed (medical/surgical)	12 (38.7%)
Other	1 (3.2%)	Not applicable	12 (38.7%)
None	10 (32.3%)		
Not reported	5 (16.1%)	Level of healthcare intervention	
		Acute care hospital level interventions	7 (22.6%)
		Jurisdiction/public health/population level interventions	24 (77.4%)

COVID-19, coronavirus disease-2019; REB, research ethics board.

Of the 31 studies (10, 27–56) reporting mortality data, 27 reported (10, 27–31, 33–41, 43–46, 48, 50–56) on all-cause mortality. Another 16 studies (10, 27, 28, 30, 31, 33–35, 38, 41, 47, 51–53, 55, 56) reported non-COVID-19 mortality data. There were 30 peer-reviewed publications (96.8%) (10, 27–49, 51–56) and 1 pre-print (3.2%) (10). There were 30 (96.8%) cohort observational studies (10, 27–46, 48–56), and 1 (3.2%) case-control (47) observational study with no randomized control trials. Research ethics board (REB) approval and consent were obtained in 6 (19.4%) studies (29, 36, 37, 45, 50, 53), 14 (45.2%) studies (10, 32, 35, 38–44, 47–49, 54) stated formal ethics approval was not required or the study was exempted while 11 (35.2%) studies (27, 28, 30, 33, 34, 46, 51, 52, 54–56) did not report REB approval or were not applicable (stated as none with no explanation). The setting for these studies were either acute care hospitals (7 studies, 22.6%) (37, 40, 42, 44, 47, 49, 50) or jurisdictional or national studies (24 studies, 77.4%) (10, 27–36, 38, 39, 41, 43, 45, 46, 48, 51–56). Nearly half of the studies (15 studies, 48.4%) had no funding or no funding declared (27, 31–35, 37, 38, 42, 46–48, 52, 55, 56). The majority of studies were performed in a single country (28/31 studies, 90.3%) (10, 27, 29–48, 50–53, 55, 56). There were no missing data from the studies included in the meta-analysis.

### Risk of bias

Supplementary Tables 4A, B demonstrate RoB for cohort and case-control studies, respectively (using the NOS).

For cohort studies (Supplementary Table 4A), full scores for NOS were found in only 1/30 studies (3.3%) (37). Common deficiencies were demonstrated in the following areas: lack of adequate length of follow-up (13 studies, 43.3%), lack of proper follow-up overall (19 studies, 63.3%), and lack of cohort comparability (19 studies, 63.3%).

For case-control studies (Supplementary Table 4B), 1/1 study (100%) had a full NOS score (47).

### Data synthesis and analysis

#### Primary and secondary outcomes and GRADE assessments

Supplementary Table 3 shows study outcomes, which demonstrated significant changes in mortality (primary outcome). GRADE assessment is shown in Supplementary Table 5. Supplementary Table 6 further summarized outcomes from Supplementary Table 3 in terms of demographics. There were increases in mortality, which disproportionally affected older individuals, males, racial minorities, those with comorbidities, and those with lower socioeconomic status.

**FIGURE 2:**
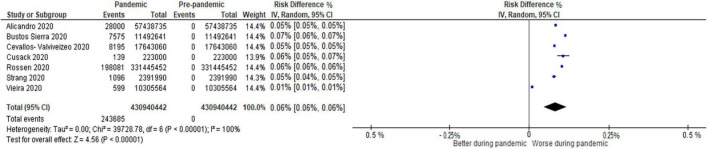
Forest plot for COVID-19 mortality. CI, confidence intervals; COVID-19, coronavirus disease 2019; IV, inverse variance.

**FIGURE 3:**
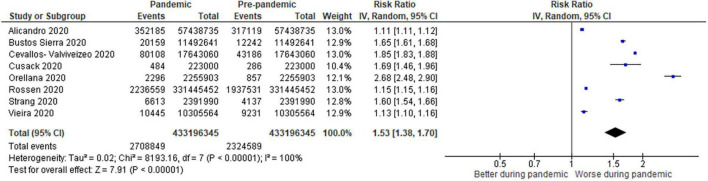
Forest plot for all–cause excess mortality. CI, confidence intervals; COVID-19, coronavirus disease 2019; IV, inverse variance.

**FIGURE 4:**
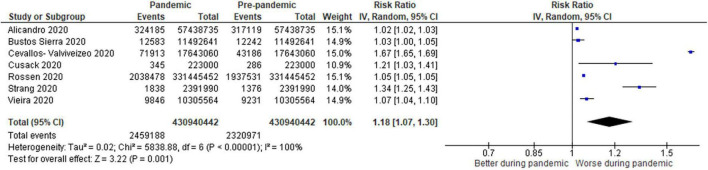
Forest plot for non-COVID-19 mortality. CI, confidence intervals; COVID-19, coronavirus disease 2019; IV, inverse variance.

Overall, 31 studies reported mortality comparisons between the COVID-19 pandemic and non-pandemic times, with 7 studies with sufficient data for meta-analysis for COVID-19 and non-COVID-19 mortality and 8 studies for all-cause mortality. These Forest plots are presented in Figures 2–4. For COVID-19 mortality (Figure 2), 7 studies (243,685 deaths out of 430,940,442 patients) were included in a meta-analysis, which demonstrated a significant increase in mortality [risk difference (RD): 0.06, 95% CI: 0.06–0.06%, *p* < 0.00001, τ^2^ = 0.0, *I*^2^ = 100%, “very low” certainty], as a proportion of the total population. This analysis was completed to quantify the COVID-19 mortality impact on the population and hence represented as a risk difference.

For all-cause mortality (Figure 3), 8 studies (0.64% pandemic mortality vs. 0.54% pre-pandemic mortality) also demonstrated a significant increase in all-cause mortality when compared to baseline pre-pandemic mortality (RR: 1.53, 95% CI: 1.38–1.70, *p* < 0.00001, τ^2^ = 0.02, *I*^2^ = 100%, “very low” certainty).

For non-COVID-19 mortality (Figure 4), 7 studies (0.57% pandemic mortality vs. 0.54% pre-pandemic mortality) showed significant increases in non-COVID-19 mortality during the pandemic when compared to baseline pre-pandemic mortality (RR: 1.18, 1.07–1.30, *p* < 0.00001, τ^2^ = 0.02, *I*^2^ = 100%, “very low” certainty).

#### Subgroups

Supplementary Figure 1 shows a pre-specified subgroup Forest plot for mortality examining HIC vs. LMIC countries. A significant increase in all-cause mortality was shown in both HIC and LMIC during the COVID-19 pandemic as compared to non-COVID-19 pandemic historical controls. However, there was a greater increase in all-cause excess mortality in LMIC (RR 2.22, 95% CI: 1.55–3.19; *p* < 0.00001, τ^2^ = 0.07, *I*^2^ = 99%, “very low” certainty) as compared to HIC (RR 1.33, 95% CI: 1.26–1.41; *p* < 0.00001, τ^2^ = 0.0, *I*^2^ = 100%, “very low” certainty). The other subgroups were not subject to meta-analysis due to a lack of data.

#### Publication bias

Visual inspection of funnel plots was not formally assessed due to the limited number of studies (<10 studies) that can be used in the meta-analysis. The diagrams can be found in Supplementary Figures 2A–C which suggest the potential for publication bias due to the asymmetry of the plot.

## Discussion

This SR demonstrates the COVID-19 pandemic likely had increased excess all-cause mortality over and above attributable deaths from COVID-19 mortality alone, indicating non-COVID-19 mortality was also likely affected, although the strength of the evidence is very uncertain. In subgroup analyses, LMIC also had higher all-cause excess mortality as compared to HIC, demonstrating further inequities between rich and poor countries. Overall, all-cause mortality and non-COVID-19 mortality increased during the pandemic when compared to historical controls, although the certainty in the level of evidence is very low (the majority of the included observational studies had high RoB) and high heterogeneity makes any conclusions very uncertain.

During the pandemic, differences in COVID-19 mortality rates were found depending on location, case mix, population structure, health system responses, and capacity, alongside individual jurisdictional healthcare policies (e.g., lockdowns, vaccine access, etc.) (7, 57–60). Early during the pandemic, only hospitalized patients could have confirmatory testing for infection (61), which caused underreporting of COVID-19 cases by a factor of 2–3 times (62), with estimated COVID-19 deaths up 6–10 times higher than officially reported (63, 64). This underestimation was magnified in subsequent waves of COVID-19 variants, due to cases outstripping testing capabilities (65, 66), even in HIC (67). For this reason, it may be beneficial to contextualize COVID-19 mortality alongside all-cause excess mortality, which has been promoted prior by other authors (68, 69). This would allow a greater understanding of the full scope and impact the pandemic has had on COVID-19 and non-COVID-19 patients alike. The use of all-cause mortality in conjunction with COVID-19 mortality may serve to be a more effective metric, emphasizing the need to monitor all-cause excess mortality alongside COVID-19 mortality, and perhaps allow prioritization of resources to prevent excess avoidable deaths.

This SR reinforces the degree and scale of unintended consequences of the COVID-19 pandemic, adding to growing literature demonstrating the impact on all-cause excess and non-COVID-19 mortality (7, 10, 28, 30, 33–35, 43, 51–53, 69), in addition to COVID-19 mortality. The COVID-19 pandemic has already surpassed the death toll of many major world events (e.g., World War II 1939, H1N1 2009), and only outpaced by larger plagues of the past (e.g., 1918 Spanish Flu) (70). The impact of COVID-19 on mortality is highly stratified by country. Certain countries (e.g., India, USA, Brazil) had the highest excess mortality, while others (e.g., Australia, New Zealand, Taiwan, Singapore) have reported negative excess mortalities (69), likely due to their COVID-19 containment strategies and differences in healthcare resource allocation with decreased non-COVID-19 mortality from their populations staying home. Each jurisdictional strategy (e.g., COVID-19 elimination vs. mitigation, access to vaccinations and medications, demographic and geographic factors) has likely led to discrepancies in the global impact of all-cause excess mortality alongside COVID-19 mortality (7), which has varied throughout different waves of the pandemic (28, 69). The continued pandemic has had profound effects on the health of non-COVID-19 patients (7, 71), as pandemic patients have utilized resources, caused invoking of pandemic control measures, and disrupted care throughout the entire healthcare system (7). Furthermore, there are likely lasting impacts on our healthcare system (72, 73), and on those who have valiantly served during the pandemic, including mental health effects for patients and healthcare workers (72, 74, 75). There are high reported rates of burnout from the COVID-19 pandemic (74), particularly in frontline workers (75), which has led to attrition and further exacerbated staffing shortages (76), from which the healthcare system may never fully recover. This could further exacerbate all-cause excess mortality.

Our strengths include rigorous adherence to SR methodology, consisting of: (1) broad eligibility criteria (2) study selection by independent adjudicators using *a priori* criteria to minimize selection bias; and (3) comprehensive search strategy to minimize publication bias. Data abstraction and critical appraisal were conducted independently and in duplicate from established PRISMA recommendations. We performed a rigorous assessment of study quality using NOS tools for observation cohort/case-control studies. Moreover, we performed rigorous assessment and summation of the level of certainty using GRADE. While mostly observational in nature, the meta-analyzed studies included rely primarily on government databases that cover multiple jurisdictions and millions of people.

This SR has several limitations, the majority of which relate to the outcomes reported in the studies. Consequently, only a subset of the studies with large population-based data was included in the meta-analysis for COVID-19 mortality, all-cause mortality, and non-COVID-19 mortality. Other studies were excluded from our meta-analysis if they had incomplete data, duplicate data from overlapping jurisdictional areas in which we selected the study with the most participants, or if they contained mortality data in formats that could not be meta-analyzed. Our heterogeneity was high, which is unsurprising given the initial spread of COVID-19, testing capabilities, economic disparity, the ranges of policy responses from across the world, and diverse populations which all could have impacted the number of deaths recorded (77). All outcomes’ GRADE certainty of evidence was very low, precluding definitive conclusions from being drawn. All the studies included were observational in nature and GRADE certainty was downgraded due to a high proportion of the studies with high RoB. In addition, the calculation of non-COVID-19 mortality can be skewed by a lack of testing.

## Conclusion

The COVID-19 pandemic has resulted in all-cause excess mortality greater than the number that can be accounted for by increases due to mortality from COVID-19 alone. Monitoring all-cause excess mortality may help health policy decision-makers and governments alike understand the true overall impacts of the COVID-19 pandemic on mortality.

## Data availability statement

The original contributions presented in this study are included in the article/Supplementary material, further inquiries can be directed to the corresponding author.

## Author contributions

DL, OR, SB, HS, DZ, MS, KL, HC, SD, PG, BM, AV, BK, DC, AA, DN, KF, and VL have assisted with drafting this manuscript, alongside critical revisions for intellectual content, made substantial contributions to: design and/or development of this SR, data acquisition, meta-analysis and data interpretation, and gave final approval of the published version of this manuscript (Supplementary Appendix 2). All authors contributed to the article and approved the submitted version.
