# Supplementary material for: Coronavirus disease 2019 (COVID-19) excess mortality outcomes associated with pandemic effects study (COPES): A systematic review and meta-analysis
Source: Front Med (Lausanne). 2022 Dec 16;9:999225. doi: 10.3389/fmed.2022.999225 (PMC9800609; doi:10.3389/fmed.2022.999225)
Supplement: Supplementary file 2 [file Data_Sheet_2.docx]

**Supplemental Appendix 2:** Detailed breakdown of author contributions

Conception: Lau (VL), Lu (DL), Rewa, Bagshaw, Sebastianski (MS)

Background: Lau, Lu, Rewa, Bagshaw, Sebastianski, Fiest, Niven, Stelfox, Zuege

Design: Lau, Lu, Rewa, Bagshaw, Sebastianski, Stelfox, Zuege

Acquisition of data: Lau, Sebastianski, Dhanoa (SD), Cheema (HC), Lewis (KL), Geeraert (PG), Lu, Merrick (BM), Vander Leek (AVL), Kula (BK), Chaudhuri (DC), Agrawal (AA)

Analysis of data: Lau, Lu, Dhanoa, Cheema, Lewis, Geeraert, Merrick, Vander Leek, Sebastianski, Kula, Chaudhuri, Agrawal, Niven, Fiest, Stelfox, Zuege, Rewa, Bagshaw

Drafting the manuscript: Lu, Lau, Dhanoa, Cheema, Lewis, Geeraert, Merrick, Vander Leek, Sebastianski, Kula, Chaudhuri, Agrawal, Niven, Fiest, Stelfox, Zuege, Rewa, Bagshaw

Revising the manuscript: Lau, Lu, Dhanoa, Cheema, Lewis, Geeraert, Merrick, Vander Leek, Sebastianski, Kula, Chaudhuri, Agrawal, Niven, Fiest, Stelfox, Zuege, Rewa, Bagshaw

Librarian: Keto-Lambert (DKL)
