# Supplementary figures and images for: Coronavirus disease 2019 (COVID-19) excess mortality outcomes associated with pandemic effects study (COPES): A systematic review and meta-analysis
Source: Front Med (Lausanne). 2022 Dec 16;9:999225. doi: 10.3389/fmed.2022.999225 (PMC9800609; doi:10.3389/fmed.2022.999225)

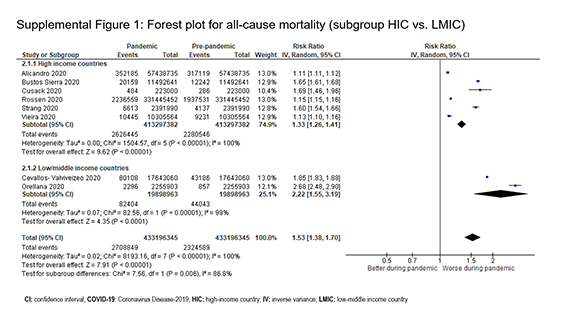

Supplement: Supplementary file 3 [file Image_1.TIF]

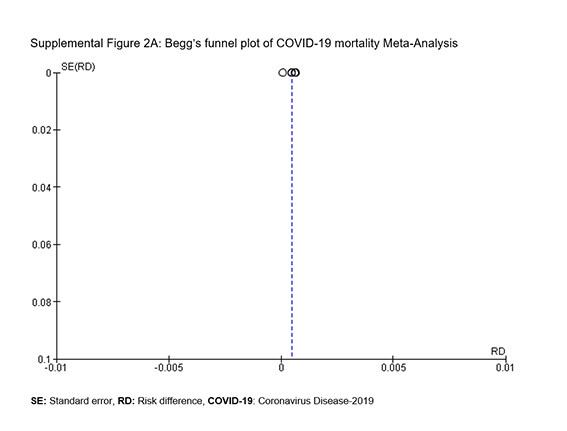

Supplement: Supplementary file 4 [file Image_2.TIF]

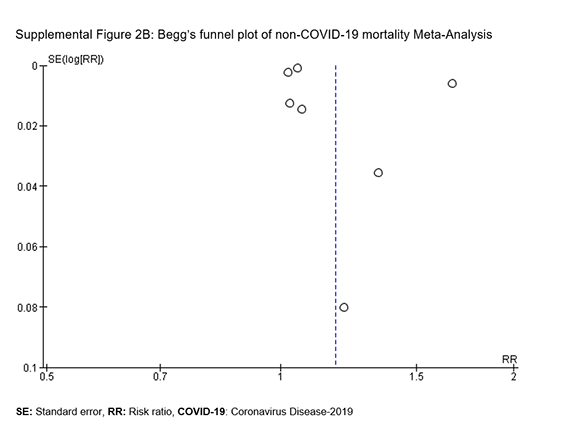

Supplement: Supplementary file 5 [file Image_3.TIF]

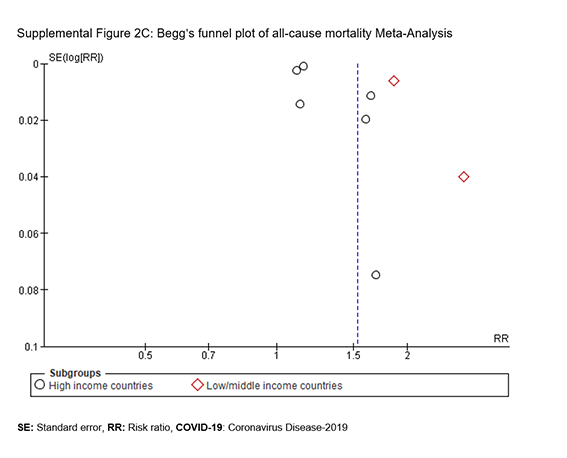

Supplement: Supplementary file 6 [file Image_4.TIF]
