# Supplementary material for: Coronavirus disease 2019 (COVID-19) excess mortality outcomes associated with pandemic effects study (COPES): A systematic review and meta-analysis
Source: Front Med (Lausanne). 2022 Dec 16;9:999225. doi: 10.3389/fmed.2022.999225 (PMC9800609; doi:10.3389/fmed.2022.999225)
Supplement: Supplementary file 8 [file Table_2.docx]

**Supplemental Table 2: Characteristics of COVID-19 mortality related papers (pre-pandemic vs. pandemic periods)**

| **Study Author (year)** | **Study Design** | **Country** | **Covid-19 Mortality Reported** | **All-cause mortality Reported** | **Non-COVID-19 Mortality Data Reported** | **Included in Meta-Analysis** | **Pandemic period** | **Non-pandemic period** |
| --- | --- | --- | --- | --- | --- | --- | --- | --- |
| Alicandro 2020 | Observational (cohort) | Italy | Yes | Yes | Yes | Yes | Jan 1 – Jun 30, 2020 | Jan 1 – Jun 30, 2015-2019 |
| Bilinski 2020 | Observational (cohort) | Multinational | Yes | Yes | Yes | No | Feb 13 – Sep 19, 2020 | Corresponding weeks from 2015-2019 |
| Birkmeyer 2020 | Observational (cohort) | USA | No | Yes | No | No | Feb 2 - Jul 11, 2020 | Corresponding weeks from 2019 |
| Blangiardo 2020 | Observational (cohort) | Italy | Yes | Yes | Yes | No | Jan 1 - Apr 28, 2020 | Corresponding weeks from 2016 - 2019 |
| Bustos Sierra 2020 | Observational (cohort) | Belgium | Yes | Yes | Yes | Yes | Mar 10 – Jun 21, 2020 | January 1900 - June 2020 |
| Cates 2020 | Observational (cohort) | USA | Yes | No | No | No | Mar 1 – May 31, 2020 | Oct 1, 2018 – Feb 1, 2020 |
| Cevallos-Valdiviezo 2020 | Observational (cohort) | Ecuador | Yes | Yes | Yes | Yes | Mar 17 – Oct 22, 2020 | Jan 1, 2014 – Mar 16, 2020 |
| Conti 2020 | Observational (cohort) | Italy | Yes | Yes | Yes | No | Jan 1 - Apr 30, 2020 | Jan 1 – Apr 30, 2019 |
| Cusack 2020 | Observational (cohort) | Ireland | Yes | Yes | Yes | Yes | Mar 11 – Jun 30, 2020 | Jan 1, 2015 – Jun 30, 2019 |
| Friedman 2020 | Observational (cohort) | Mexico | Yes | Yes | No |  | Apr 14 - May 11, 2020 | Jan 1 2014 - Dec 31, 2019 |
| Habonimana 2020 | Observational (cohort) | Burundi | Yes | Yes | No | No | Jan 1 – May 30, 2020 | Jan 1 – May 30, 2019 |
| Jacobson 2020 | Observational (cohort) | USA | Yes | Yes | Yes | No | Mar 1 - May 30, 2020 | 2018 weekly data (2019 census) |
| Lerner 2020 | Observational (cohort) | USA | No | Yes | No | No | Week 10 (Mar 2-8) vs Week 16 of 2020 compared (call volume) [also compared to previous year of the same time frame)  Week 11 (Mar 9-15) vs Week 15 of 2020 compared (proportion of death)  Week 10 vs. week 13 2020 (actual EMS activation and % potential injury reported is compared) | 40th week to 21st of next year (2017-2020) |
| Little 2020 | Observational (cohort) | England | Yes | Yes | No | No | Mar 1 - Apr 30, 2020 | Mar 1 to Apr 30, 2019 |
| Mannucci 2020 | Observational (cohort) | Italy | Yes | Yes | Yes | No | Feb 20 - Mar 31, 2020 | Feb 20 to Mar 31, 2015-2019 |
| McGuinness 2020 | Observational (cohort) | USA | Yes | No | No | No | Mar 1 - Apr 6, 2020 | Feb 1, 2016 - Feb 1, 2020 |
| Michelozzi 2020 | Observational (cohort) | Italy | Yes | Yes | No | No | Northern Italy: Feb 29 - Apr 20, 2020.  Central and southern Italy: Mar 11 - Apr 20, 2020 | Northern Italy: Dec 1, 2019 - Feb 29, 2020.  Central and Southern Italy: Dec 1, 2019 - Mar 11, 2020 |
| Miles 2020 | Observational (cohort) | England and Wales | Yes | Yes | No | No | Up to May 13, 2020 | Up to May 13, 2020 |
| Nef 2020 | Observational (cohort) | Germany | No | Yes | No | No | Mar 23 - Apr 26, 2020 | Mar 23 - April 26, 2019 |
| Orellana 2020 | Observational (cohort) | Brazil | Yes | Yes | No | Yes | Epidemiological Week 12 to 17, 2020 | Epidemiological Week 12 to 17, 2019 and 2018 |
| Perkin 2020 | Observational (case-control) | UK | Yes | Yes | No | Yes | 6-week period following March 12, 2020 | 6-week period following March 12, 2019 |
| Piccininni 2020 | Observational (cohort) | Italy | Yes | No | No | No | Feb 21 – Apr 11, 2020 | Jan 1, 2012 – Jan 1 2020 |
| Richards-Belle 2020 | Observational (cohort) | UK | Yes | Yes | No | Yes | Feb 1 - Aug 31, 2020 | Jan 1 - Dec 31, 2017 - 2019 |
| Riley 2020 | Observational (cohort) | UK | Yes | Yes | Yes | No | Apr 1 - Apr 30, 2020 | Apr 1 - Apr 30, 2019 |
| Rossen 2020 | Observational (cohort) | USA | Yes | Yes | Yes | Yes | Jan 26 - Oct 3 2020 | Same weeks as pandemic period (2015-2019) |
| Saglietto 2020 | Observational (cohort) | Italy | Yes | Yes | Yes | No | Feb 23 - Mar 21, 2020 | Feb 23 - Mar 21, 2015-2019 |
| Stokes 2020 | Observational (cohort) | USA | Yes | Yes | Yes | No | Feb 1 – Sep 23, 2020 | Feb 1 – Sep 23, 2013-2018 |
| Strang 2020 | Observational (cohort) | Sweden | No | Yes | No | Yes | Mar 1 - May 30, 2020 | Mar 1 - May 30, 2016-2019 |
| Vestergaard 2020 | Observational (cohort) | EU | Yes | Yes | Yes | No | Weeks 1-18, 2020 | Weeks 1-18, 2016 - 2019 |
| Vieira 2020 | Observational (cohort) | Portugal | Yes | Yes | Yes | Yes | Mar 16 - Apr 14, 2020 | Jan 1 – Apr 14, 2010 - 2019 |
| Woolf 2020 | Observational (cohort) | USA | Yes | Yes | Yes | No | Mar 1 - Apr 25, 2020 | Dec 29, 2013 - Feb 29, 2020 |
