# Supplementary material for: Coronavirus disease 2019 (COVID-19) excess mortality outcomes associated with pandemic effects study (COPES): A systematic review and meta-analysis
Source: Front Med (Lausanne). 2022 Dec 16;9:999225. doi: 10.3389/fmed.2022.999225 (PMC9800609; doi:10.3389/fmed.2022.999225)
Supplement: Supplementary file 9 [file Table_3.docx]

Supplemental Table 3: Summary statistics of statistically significant outcomes for COPES COVID-19 Illness during COVID-19 pandemic

| **Study** | **Region** | **Time period** | **COVID-19 mortality** | **Absolute all cause Excess Mortality** | **Excess all-cause mortality rate** | **Comments** |
| --- | --- | --- | --- | --- | --- | --- |
| Alicandro 2020 | Italy (*mortality data from 93% of Italian municipalities)*  *95% of population (60,461,826 - 2020)* | Mar 1– Apr 30  2020  Jan 1 – Jun 30  2020 | ~28 000  NR | 46 463  35 066 (352185 vs 317119 in 2015-2019) | 50.5% in March, 38.0% in April  11.1% January – June vs (2015-2019) | 2020 started as a year of low mortality and in January and February 2020 the number of deaths was respectively 9 and 5% lower as compared to the same months of previous years. Such lower mortality along with that of June 2020 partly offset the excess mortality of March and April 2020, leading excess deaths in the first six months of 2020 |
| Bilinski 2020 | USA | Feb 13 – July 25  2020  Feb 13 – Sep 19  2020 | 145 546  198589 (60.3/100k) | 235 610  N/A | 71.6/100k excess mortality (Feb 13- Jul 25)  vs (2015-2019) | Compared with other countries, the US experienced high COVID-19–associated mortality and excess all cause mortality into September 2020. After the first peak in early spring, US death rates from COVID-19 and from all causes remained higher than even countries with high COVID-19 mortality  If the US had comparable death rates with most high-mortality countries beginning May 10, it would have had 44210 to 104177 fewer deaths (22%-52%)  In countries with moderate COVID-19 mortality, excess all-cause mortality remained negligible  Throughout the pandemic. In countries with high COVID-19 mortality, excess all-cause mortality reached as high as 102.1/ 100000 in Spain, while in the US it was 71.6/100000 |
| Birkmeyer 2020 | USA | Feb 1 – Jul 31  2020 | NR | NR | NR | If less seriously ill patients were disproportionately staying away from the hospital during the COVID-19 pandemic, we expected in-hospital mortality rates to rise.  Relative to the 2.1 percent in-hospital mortality rate for non-COVID-19 admissions during February 2020, in-hospital mortality rose by 0.3 percent (p < 0:05) in April before returning to baseline in May and June.  well below baseline for patients with pneumonia (−44 percent), chronic obstructive pulmonary disease/asthma (−40 percent), sepsis (−25 percent), urinary tract infection (−24 percent), and acute ST-elevation myocardial infarction (−22 percent)  suggested that much of the increase in in-hospital mortality for non-COVID admissions during April occurred for patients living in majority-Black, majority-Hispanic, or high poverty ZIP codes  In April, mortality in this subgroup was 0.5 percent higher than for those not in the subgroup (p < 0:03). By the end of May, that disparity in mortality had disappeared period, admissions declines varied only modestly by patient demographic factors, including insurance status and minority and income characteristics of the areas in which patients lived.  Hospital, in-hospital mortality in patients without COVID-19 increased only modestly during the nadir of medical admissions in April before returning to pre- COVID-19 levels in June. Largely flat mortality rates imply that the total number of in-hospital deaths for non-COVID-19 medical conditions declined by nearly the same degree as admissions.  The increased in-hospital mortality rate during the nadir in April for non-COVID- 19 medical admissions was more pronounced for patients residing in minority or poor neighborhoods. There is evidence that Black patients  diagnosed with COVID-19 were more likely to be admitted to the hospital and to experience higher in-hospital mortality (in regards to admission info) largely null findings in this regard may simply reflect that the pandemic affected patients’ decision making in a powerful, universal way that trumped the usual determinants of health care use.21  Less consistent with the elasticity view are the continued lower admissions for acute ST-elevation myocardial infarction and stroke, generally considered to be acute nondiscretionary admissions |
| Blangiardo 2020 | Italy (North West, Lombardia, North East, Centre, South + Islands) | Mar 1 – Apr 30 2020 | 27 938 | 41030 | 136 420 vs. 95390 (2016 – 2019) | Used model taking in account air temperature, sex, etc. using 2016-2019 data to predict 2020 excess mortality  Mortality rates for males in the North-West and the North-East regions start to deviate substantially from the estimated trend a week after Lombardia (starting 4 March), while the Centre shows a small deviation from the week starting with 11 March and the South does not show substantial differences from the model estimates of the time trend  For females there is a lagged effect of the pandemic, with mortality rates deviating from the expected trend on the week of 4 March in Lombardia and 11 March in the North-East and the North-West Found geographical differences:  City of Piacenza (in Emilia-Romagna) is the first to reach the peak of the pandemic on the week starting on 11 March, when the observed mortality rates reach 177 per 100,000 residents, corresponding to an excess mortality of 86.9% (77.5% to 94.4%). Bergamo and Pesaro (in the region of Marche, in Central Italy) peak one week later, with mortality rates of 182 and 134 per 100,000, corresponding to an excess mortality of 88.9% (81.9% to 95.2%) and 84.2% (73.8% to 93.4%).  On the other hand, the Centre and the South of Italy do not show evidence of increased net mortality |
| Bustos Sierra 2020 | Belgium  N= 11,492,641 (as of Jan 1, 2020) | Mar 10 - Jun 21  2020 | 7576 (March 20 – April 28)  *(Statistically significant period)*  9591 (Mar 10 – June 21) incl. LAB, CT, possible cases | 7917 – excess death (Mar 20- Apr 28)  *(Statistically significant period)*  20159 vs 12242 (baseline) | 64.7% (March 20 – April 28)  *(Statistically significant period)* | Correlation between the daily numbers of all-cause mortality and COVID-19 mortality was 94% (Spearman’s rho, p < 0,01)  Four weeks after the start of the lockdown, on April 8th, the peak of COVID-19 mortality was reached with a daily total of 321 deaths. Two days later, a peak of all cause mortality was observed (April 10th: 669 deaths).  Period of statistically significant excess mortality was observed from March 20th to April 28^th^. There was no significant excess mortality between April 29th and May 4th. Subsequently, three additional days of significant excess mortality were observed: May 5th, 8th, and 9th.  In this period (Mar 20 – Apr 28), the number of excess deaths from all causes was almost equal to the number of COVID-19 deaths (7576), indicating that 96% of the excess mortality were likely attributable to COVID-19 or to the health crisis it created.  2015–19, Belgium had a seasonal mortality pattern with an average of 321 deaths per day during winter (weeks 41 to 19) against 270 during summer (weeks 20 to 40). The highest number of daily deaths in this period was recorded on March 7th 2018, at 465 deaths, coinciding with the peak of the ongoing flu epidemic  1) a substantial statistically significant excess mortality, 2) a high correlation between COVID- 19 mortality and excess all-cause mortality 3) surveillance of laboratory-confirmed COVID-19 deaths alone would have underestimated the number of COVID-19- related deaths by approximately 30%.  During 21 days (between March 27th and April 17th), over 500 deaths per day were observed, including seven days with over 600 deaths. Belgium observed 15,398 deaths from all causes for April 2020 compared to an average of 8854 deaths for the same month in 2015–2019.  number of deaths per day are available since 1989. Between 1989 and 2020, the only other event in which  Belgium experienced more than 500 deaths per day was during the 1989 influenza A(H3N2) epidemic, with two days in mid-December with 516 and 520 deaths  The number of deaths in April 2020 was also similar to that of the flu epidemics of January 1951 and February 1960, which claimed about 15,500 deaths per month [17, 18]. These numbers have only been surpassed by the number of deaths recorded at the beginning and towards the end of the Second World War (May 1940, 23, 106 deaths; January 1945, 15,950 deaths)  The monthly mortality rate in April 2020 in Belgium (134 per 100,000) was equal to the rate of the 1989 influenza epidemic, close to the 1968–1970 Hong Kong influenza rate (147 per 100,000 in January 1970), but lower than those observed at the start and the end of the Second World War (respectively 279 and 191 per 100,000) and during the Spanish influenza pandemic. The average monthly mortality rate in 1918 was 174 per 100,000 inhabitants.  Discussions:  The inclusion of deaths in possible cases and deaths occurring outside hospital settings in our surveillance resulted in a recorded number of COVID-19 deaths very close to the number of excess deaths calculated by Be-MOMO.  with difficulties in access to care during lockdown and with patients hesitating to seek medical care for fear of catching the virus. Increased frailty [23] and downward spiral syndrome (or geriatric failure to thrive) in the elderly, as a result of social isolation and reduced physical activity, may have also played a key role  Not all deaths related to the COVID-19 can be considered excess mortality, as a small portion of these deaths would likely have occurred at that time as part of the expected number of deaths. But no short-term harvest effects (a statistically significant lower number than expected daily deaths) were observed after the peak, suggesting that the majority of those who died would not have died in the short term.  Belgium’s COVID-19 mortality rate would be reduced by 57% if reporting had been limited to deaths in laboratory confirmed hospitalised cases. Moreover, the COVID-19 mortality surveillance that was used in the country contributed to the identification of the burden of COVID- 19 infection in NHs. At the same time, the underestimation of the number of COVID-19 cases in the general population related to the initially conservative testing strategy, has created doubt in the media and policymakers about the accuracy of the extremely high figures of COVID-19 deaths in Belgium, especially given the negative impact on the country’s image with economic and diplomatic consequences.  With an alternative and more restricted definition, Belgium would have attracted less negative attention  from the international community, but the alarming situation in NHs would likely not have been picked up.  We believe that the implementation of a specific COVID-19 mortality monitoring system in the country, including possible cases and deaths in long-term care facilities, has proven to be appropriate to allow a proper assessment of the impact of COVID- 19. specificities have led to misleading international comparisons, and differences in country methods for mortality data collection should be better communicated. Although the monthly number of deaths in April 2020 is exceptionally high, ranking the COVID- 19 epidemic as a fatal event, the monthly mortality rate was lower than during other major fatal events of the last century. |
| Cates 2020 | USA (national veterans health administration VHA) | Mar 1 – May 31  2020 | percentage of COVID-19 patients who died while hospitalized (N= 828/3948 21.0%) | N/A | N/A | Percentage of COVID-19 patients who died while hospitalized (21.0% N= 828/3948) was more than five times that of influenza patients (N = 190 / 5453 3.8%)  Although previous analysis of VHA data found no differences in COVID-19 mortality by race/ethnicity (*4*), in this analysis, Black, Hispanic, and non-Hispanic patients. of other races had higher risks for sepsis and respiratory, neu­rologic, and renal complications than did White patients |
| Cevallos-Valdiviezo | Ecuador  (2020 WorldBank  N = 17,643,060 | Mar 17 – Oct 22  2020 | 8195 (confirmed), 4305 (probable) | 36 922 (32314-42696 95%CI)  80108 vs 43186 (baseline) | 80 108 all cause deaths | Expected deaths were estimated for the period March 17–October 22, 2020 from forecasts of an ARIMA model of order (3,0,1) with drift which was applied to daily mortality data for the period from January 1, 2014 to March 16, 2020  Estimates of excess mortality might provide a better approximation of the true COVID-19 death toll. These estimates might capture not only deaths directly attributable to the COVID-19 pandemic but also deaths from other diseases that resulted from indirect effects of the pandemic.  Note also that the levels of estimated excess mortality in general exceed those of the number of confirmed and probable COVID-19 deaths. This difference may represent deaths directly attributable to COVID- 19 that were not officially reported but also deaths from other diseases that resulted from indirect effects of the pandemic, such as the fear of being treated in hospitals overwhelmed with COVID- 19 patients or a shortage of medication, physicians, intensive care unit beds, ventilators, or a general lack of access to healthcare services. |
| Conti 2020 | Italy (health protection agency for Bergamo and Brescia)  (Brescia – 1097648, Bergamo – 1163243) | Jan 1 – Apr 30  2020 | 2973 (Bergamo), 2295 (Brescia) | 5740 (5552-5936 95%CI) [Bergamo], 3703 (3535 – 3877 95%) [Brescia]  5719 (Bergamo) [5556-5892 95%CI], 3820 (Brescia) [3676 – 3967 95%CI]  (if using March and April only) | 17 099 vs 7592 in 2019  2.55-fold (95% CS 2.50–2.61) and  1.93 (95% CS 1.89–1.98) increase in the number of deaths in Bergamo and Brescia respectively | Based on these time-series, and considering that the population of each HPA remained stable from 2019 to 2020 (HPA Brescia: from 1 089 602 to 1 097 648; HPA Bergamo: from 1 160 374 to 1 163 243), we computed the expected daily number of deaths for 2020 as the average daily number of deaths that occurred in 2019.  Overall, the daily average number of deaths rose significantly from 63 (SD: 10.7) to 141 (SD: 129.8), with a steeper wave of the cumulative time-series analysis in the area of Bergamo  (figure 1)  Standardized Mortality ratio  The overall daily estimated SMR peaked at 9.4 (95%CI 9.1–9.7) in Bergamo province and at 5.6 (95% CI 5.4–5.8) 46in Brescia. The highest SMRs were observed among the elderly, peaking in the age-class 70–79: 12.7 (95% CI 11.9–13.5) in Bergamo; 7.3 (95% CI 6.8–7.9) in Brescia.  in the age-class 70–79, the number of cumulative deaths increased by 5.05-fold (95% CS 4.83–5.28), compared to 2.18-fold (95% CS 1.96–2.44) among the youngest  the excess death wave started earlier in the area of Bergamo, roughly 1 week after the detection of the first COVID-19 case in Lombardy (February 21, 2020), and a few days later in the area of Brescia. In both areas, the excess death wave started before the introduction of lockdown measures (March 7, 2020), and the peaks were observed between March 20 and March 25, 2020, roughly 2 weeks after the introduction of lockdown measures,  returning to pre-epidemic levels by the end of April.  Discussions  we estimated 9443 excess deaths in the areas covered by the HPAs of Bergamo and Brescia, corresponding to a 2.2-fold increase in number of excess deaths as compared to those recorded in the same period of 2019, with a trend that showed a rapid rise starting 10 days after the first case of COVID-19 in the region after the beginning of the disease outbreak, in line with preliminary findings from March only that the overall mortality did not increase in Italian provinces with low rates of infection [13]  The discrepancy might be due to a lower infection rate among the oldest, the mobility and social contacts of whom are frequently reduced  2020, particularly March and April, indicating that the COVID-19 outbreak had a substantially larger  impact than official estimates. Time-series analyses suggest that the national and local restrictions had a  massive effect, providing a considerable reduction of COVID-19 burden (this probably based on cases reducing following lockdowns) |
| Cusack 2020 | Ireland (District of Kildare) N= 223 000 | Mar 1 - Jun 30  2020 | 139, (113 in nursing homes) | 198 Total  131 for nursing and residential home)  January to June | 484 (69% excess) vs. 286 (2015-2019) p=0.69 ,  293 (81% excess) vs. 162 (2015-2019) nursing home and residential home)  January to June | The peak excess total death percentage was 359% in April 2020, commencing with a small excess in March (30%), continuing into May (63%) and falling again in June (37%). In the nursing and residential home setting those excess death per-centages were most marked at 527% in April, with 27% in March, 54% in May and 17% in June. Underlying medical conditions were recorded in 99% of those dying from Covid-19 and the average age of the deceased was 82.5 years with median of 78 years and 55% of those dying were female and 45% male.  there were no excess deaths calculated in this study for January to February 2020 when compared with 2015–2019  Excess deaths notiﬁed to the Coroner in March to June 2020 were greatest in April at 359%. The excess commenced at a lower level from the latter half of March (30%) and then continued after the April peak into May (63%) and June (37%), with an overall 117% for March to June, as illustrated in non-natural cause deaths and Covid-19 deaths were subtracted for each of the months to give the ﬁgures for natural non- Covid-19 deaths with excess of 27%, 100%, 18% and 18% for March, April, May and June compared to 2015–2019  unexplained residual excess of 60 deaths out of those remaining due to natural causes (overall 38% residual excess compared to the 2015–2019 such deaths  figures reflecting the larger female population in nursing and residential homes. There was a small excess of non-Covid-19 natural deaths notified from nursing homes in March 2020 (17%) compared with a large excess in April 2020 (118%) but none recorded in May or June 2020    Of the 139 cases notified, 137 (99%) had underlying conditions: 80 (58%) cardiovascular (including hypertension); 78 (57%) dementia; 30 (22%) respiratory; 19 (43%) neurological; 17 (12%) oncological; 15 (11%) diabetes; and 9 (7%) renal  in whom lethargy, withdrawal, sudden general deterioration and gastrointestinal symptoms became recognised as clinical presentations as the pandemic developed. In addition, loss of smell, loss of taste and distortion of taste were also only later recognised as classical symptoms.4 These less classical symptoms and later recognised symptoms may have led to underdiagnosis of Covid-19 in the older population in the initial periods.  final diagnosis of Covid-19 is an assessment of clinical probability based on the case criteria and all the clinical information and then becomes the confirmed certified cause of death  medical caution must be added relating to later complications from the infection causing later morbidity and mortality in persons who have apparently recovered from the Covid-19 infection but who develop post viral pathology in major organs such as the lungs, heart and kidneys and also neurological sequelae which will be another source of delayed morbidity and excess mortality |
| Friedman | Mexico (Tijuana) | Apr 14 – May 11 vs (Jan 1 2014 - Dec 31 2019) | 80 – out of hospital death | 194.7 (135.5-253.9 95%CI) out of hospital deaths | 329 deaths (Apr 14- May 11) vs predicted baseline 134.7 (75.1 – 193.5)  145% increased (70-338% 95CI) | Using EMS data, we observed increases in out-of-hospital deaths in Tijuana that were nearly threefold greater magnitude than increases reported using EMS data in Italy. Out-of-hospital mortality was defined when patient was found dead-on-arrival, or died before reaching a hospital, as documented by EMS  On May 11th Tijuana had the highest number of COVID-19 deaths of any municipality in the country (170) and the mortality rate (17.3 per 100,000 people) was almost six times the national rate of 3.1 per 100,000 people  chief complaint of “respiratory”, “difficulty breathing”, or “respiratory infection” (which collectively represented the majority of cases) or b) a chief complaint that was metabolic or gastrointestinal in nature, combined with an SpO2 of less than 92%. (odd presentation of COVID)  sought to ensure that no difference in nomenclature, classification, or life support practices occurred in response to the onset of the COVID-19 crisis that could cause an apparent increase in out-of-hospital mortality  410 average weekly cases between April 14th and May 11th, compared to a weekly mean of 382.9 in 2019  ... Contrastingly, both urgent and deceased cases rose, reaching 11.2% and 20.0% respectively, as compared to 6.7% and 7.9% respectively in 2019.  January to March 2020, the number and proportion of out-of-hospital mortality cases was within or below the 95% prediction interval based on trends observed from 2014 to 2019  April 14th 80 deaths (Figure 3, Part A) exceeding the previously observed maximum in the timeseries (Figure 2). The 329 deaths occurring from April 14th to May 11th were compared to the predicted number of 134.7 (95%CI: 75.1-193.5) for the same period, yielding an estimated excess of 194.7 (95%CI: 135.5-253.9) deaths.  peak observed period of out-of-hospital mortality lined up exactly with the highest observed rates of COVID-19 deaths according to official statistics (Figure 3, Part C). 262 deaths among confirmed COVID-19 patients were reported during the same period. However, only 8 of these deaths were reported as occurring “in an outpatient context”, the remainder being reported as occurring among “hospitalized patients”29.  Out-of-hospital deaths during the period of April 14th to May 11th were majority men (68.4%), of working age 18-64 (64.1%), who were beneficiaries of the Mexican National Institute for Social Security (IMSS) healthcare system (45.9%) (Table 1). IMSS is a social security scheme providing health care to individuals employed in the private formal sector. Although the age and gender patterns were largely similar to those observed throughout 2019, they were more likely to be IMSS beneficiaries, (45.9% vs. 29.9%, difference=16.3% [95%CI: 10.0%-22.0%]).  Compared with respiratory patients in 2019, respiratory patients in the peak observed epidemic period more likely to be IMSS beneficiaries (66.4% vs. 38.9%, difference=27.6% [22.3%-32.9%]), and have an SpO2 lower than 90% (54.8% vs. 32.4%, difference=22.4% [17.0%-27.9%]).  clusters of respiratory cases during the peak epidemic period were most concentrated in highest-and high-SES quintiles of Tijuana. Contrastingly, the largest clusters of out-of-hospital mortality cases were seen in the low-SES quintile. As rates per 100,000 people the low-SES quintile of the population saw the highest rate of out-of-hospital mortality at 24.5, while the high-SES quintile saw the highest rate of respiratory cases, at 30.9.  Although respiratory cases were strikingly concentrated in the high- and highest-SES quintiles, the highest out-of-hospital mortality rates were observed in low-SES areas. There is a notable difference between respiratory cases and deaths, which may suggest that the profile of individuals who have the economic or social capital to seek care early for respiratory symptoms in Tijuana differs from those who do not interact with the medical system until after their death. This finding adds to a growing body of literature and social commentary suggesting that social inequalities may be translating into inequalities in the risk of infection or death from COVID-19 in numerous contexts37–43 |
| Habonimana 2020 | Burundi | Jan 1 to May 30 2020 vs 2019 | Mortality data was compared from year to year with no specific data |  |  | Burundi announced the first two COVID-19 confirmed cases on 31 March 2020. until late July 2020, only one COVID-19-related death had been officially reported earlier on 13 April 2020  no evidence in difference in mortality rates between 2019 and 2020 by age, BMI, residence, COVID-19-related symptoms and underlying health conditions  Kaplan–Meier curves indicate that death was quicker in 2020 than in 2019 (Fig. 3). In fact, deaths occurred in 6.7 days (S.D.= 8.9, median = 3) in 2020 against 7.8 days (S.D. = 10.9, median = 4) in 2019.   - we did not detect significant predictors of death with one or more COVID-19 symptom - we did not find evidence that the disease caused mortality rates to increase compared to the previous year   With a hospital death rate of nearly 13% in April, the figure sharply rose to about 22% in the following month - could be considered as an onset of the pandemic in Burundi. time-to-death was much shorter in 2020 with patients dying in 6.7 days on average  World Bank reported COVID-19 deaths occurred at younger ages in low- and middle-income countries [19], our study showed neutral stand with that regard: important to note: contradict a wealth of literature on the vulnerability of older people to COVID-19  In our study, we also found significant evidence that the likelihood of dying from one or more COVID-19 symptoms decreased with male sex and profession. This finding contradicts that of the World Bank in their study conducted on data from 26 countries which found a male vulnerability  Results from the logistic regression showed a much lower likelihood of dying with one or more COVID-19 symptom(s) among males and patients with a profession  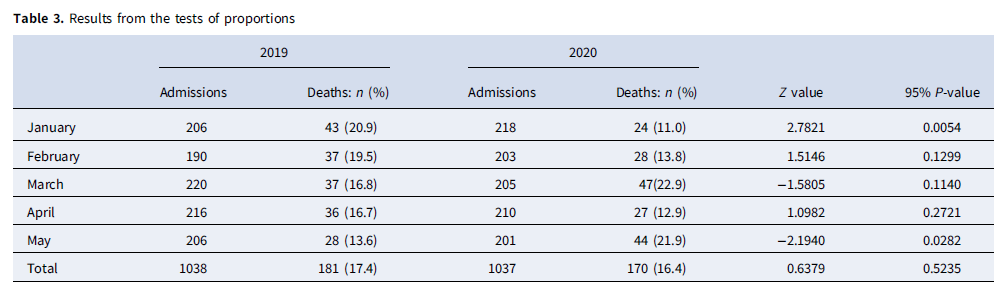 |
| Jacobson 2020 | USA | Mar 1 - May 30 2020 vs average of 2018 death (with 2019 census) | Dependent on age cohort (average weekly deaths)  Does have 2020 COVID-19 weekly death statistics across age ranges |  |  | For 17 of the 18 age and gender cohorts, the 2020 non-COVID-19 average weekly deaths are more than the hybrid 2019 average Weekly deaths. The one exception is the 5- to 14-year age group for females, which indicates that the 2020 non - COVID-19 average weekly deaths are fewer; hence, COVID-19 was protective for these young girl  For women, two age cohorts (25-34 and 35-44 years) show a statistically significant (P-value < 0.001) increase in expected 2020 non- COVID-19 weekly deaths compared with the hybrid 2019 weekly deaths.  For men, four age cohorts (15-24, 25-34, 35-44 and 45-54 years) show a statistically significant increase in expected 2020 non-COVID-19 weekly deaths for males aged 5-14, 75-84 and >85 years and for females aged 15-24, 45-54, 55-64, 65-74, 75-84 and >85 years, the expected 2020 weekly death increases may be explained by COVID-19 because increases in non-COVID-19 deaths were statistically insignificant (P> 0.05)  all but one age and gender cohort, there were more 2020 average weekly deaths than the hybrid 2019 average weekly deaths  One possible explanation for this is that 2019 weekly deaths are not uniformly distributed across the year, which is highly likely. Another explanation is that because the 2020 population has a higher is larger than 2019, there may be more deaths, although this increase is likely to be negligible compared with the actual number of deaths. To overcome these limitations, we used a P-value cut-off of 0.001 |
| Lerner 2020 | USA | Week 11 (Mar 9-15) vs Week 15 of 2020 compared (proportion of death) |  |  |  | Proportion of scene deaths nearly doubled, increasing from 1.49% to 2.77% among all EMS activations with patient contact by week 15 [vs. week 11 2020]  Raw numbers of EMS-attended scene deaths reported in 2020, the number increases from 6,294 in week 11 to 8,942 in week 15  Further, over a third of patients who delayed presenting for care of their myocardial infarction cited fear of COVID-19 or not wanting to burden the hospital as a reason for their delay. |
| Little 2020 | England (City of London - ~9 million) | Mar 1 - Apr 30 2020 vs 2019 | 21.7% (in-hospital STEMI  COVID+) vs. 9.3% non-COVID | See comments | See comments | In-hospital all-cause mortality (21.7% (10) vs 9.3% (28)) p = 0.012  Comparing 2020 vs 2019 (no delineation covid+ or not)  2020 study population to the 2019 control population revealed comparable door to balloon times between the two groups, contrary to previous reports24 with comparable in-hospital mortality rates. This suggests that modifications to the existing in-hospital PPCI pathways, such as routine use of PPE and redeployment of staff to other clinical areas, neither delayed the time taken to achieve coronary revascularisation (door to balloon time) or resulted in worse outcomes  median ischaemic time was 36 min longer in 2020 than 2019, driven predominantly by an increase in the median time from first call for help to arrival at a PPCI centre  The increased door to balloon time and subsequent increased ischaemic time for COVID-19 positive patients compared with COVID-19 negative patients in 2020 likely reflects the increased procedural complexity of these cases rather than system-related delays as all centres mandated full PPE for all PPCI cases given the unknown COVID-19 status at time of procedure  Total ischemic time is 4 min longer for COVID+ patients (360 vs 257 min p=0.008) |
| Mannucci 2020 | Italy (52,452,445 out of 60.4M accounted for) | Feb 20 - Mar 31 vs Feb 20 to Mar 31 (2015-2019) | 13710 | 26 701 | 790.0 vs 604.6 in 2019 (*100 000 inhabitants)  +27.9% difference | Non-COVID-19-Imputed Excess mortality (NCIEM) (difference between 2015-2019 mortality vs. 2020) accounting for a population of 52,452,445 (of a total population of 60,359,546)  National value of 22.2, different regions within showed correlation with NCIEM (Non-COVID-19-Imputed Excess Mortality) and COVID-19 mortality (r^2^=0.61, p<0.001), total cases (r^2^=0.3,p=0.012) and inverse with cases/total tests ratio (r^2^=0.49,p=0.001)  In some areas of the country, most notably in the Lombardia region, the capacity of hospital care, and particularly of intensive care units, was overwhelmed, possibly contributing to a very high COVID-19-related mortality rate. During study period 27.9% increase in death (data does not show cause of death) |
| mcGuinness 2020 | USA (NYU Langone Health EMR)  3837 seen in ER underwent chest imaging | Mar 1 – Apr 6 2020 vs Feb 1 2016 – Feb 1 2020 | Comparison data for risk factors provided | N/A | N/A | Total of 601 patients with COVID-19 infection, and 196 patients without COVID-19 infection requiring Invasive Mechanical Ventilation. 02/01/2016 and 02/01/2020 1,962 patients were identified with respiratory failure requiring IMV. 285 of these patients diagnosed with ARDS formed our historical pre-COVID-19 comparison cohort.  Demographics/Non mortality data:  Mechanically ventilated patients with COVID-19 infection were younger compared with the historical ARDS cohort (mean, 63 years vs 68 years; *P* , .001), but they were not different from the contemporaneous patients without COVID-19 infection (mean, 64 years). More patients with COVID-19 infection were men (426 of 601 [71%] vs 102 of 196 [52%] and 170 of 285 [60%]), compared, respectively, with the same cohorts. The cohort with COVID- 19 infection was less likely to be white non-Hispanic (234 of 601 [39%] vs 93 of 196 [47%]) or African American (69 of 601[12%] vs 34 of 196 [17%]) compared with the cohort without COVID-19 infection. On average, patients with COVID-19 infection were hospitalized for 2.8 days before IMV (range 0–20 days); patients without COVID-19 infection were hospitalized 1.8 days before IMV (range 0–20 days); and historical ARDS patients were hospitalized 3.6 days before IMV (range 0–56 days).  No survival difference existed between patients with COVID-19 infection with and without barotrauma or between the historical ARDS patients with and without barotrauma  (could be due to younger age for those with barotrauma – analysis does show that barotrauma, together with older age, are associated with mortality odds for death may be negated by the younger age in the barotrauma cohort)  There was a difference in overall survival for patients with barotrauma between the historical ARDS, COVID-19–negative, and COVID-19–positive groups (*P <*.001), with patients in the historical ARDS group showing the longest survival  Univariate analysis showed that compared with discharge, death in patients with COVID-19 infection was  only associated with older age and shorter hospitalization LOS (*P <*0.001).  Multivariate analysis showed an association of mortality in patients with COVID-19 infection with greater age (odds ratio = 1.05; P <0.001), shorter LOS (odds ratio = 0.92; *P<*.001), Hispanic ethnicity (odds ratio = 1.73; *P* = .05), as well as sustaining barotrauma (odds ratio = 2.2; *P*=0.03)  In the COVID-19 negative cohort, older age and shorter LOS were associated with mortality (*P* = .05 and .002)  historical ARDS group, only shorter LOS was associated with mortality (*P* = .001)  Binary logistic regression analysis performed using barotrauma as a dependent showed that longer  LOS and younger age was associated with sustaining barotrauma in the patients with COVID-19 infection (*P* , .001 and .038, respectively) longer LOS was identified as a risk factor for barotrauma in the historical ARDS group (*P* , .05).  incidence of barotrauma was higher in the COVID-19– positive cohort (15%) compared with the historical ARDS cohort (10%; *P* = .04); the total barotrauma rate (24% vs 11%, respectively) was also higher (*P<0*.001).  Although smoking is a risk factor for barotrauma, the majority of barotrauma patients with COVID-19 infection and half of the historical patients with ARDS in our study were never smokers.  High smoking rates in China were initially thought to contribute to severe morbidity in early COVID-19 reports; however, smoking was not associated with an increased risk of hospitalization or critical illness in a large study from our institution  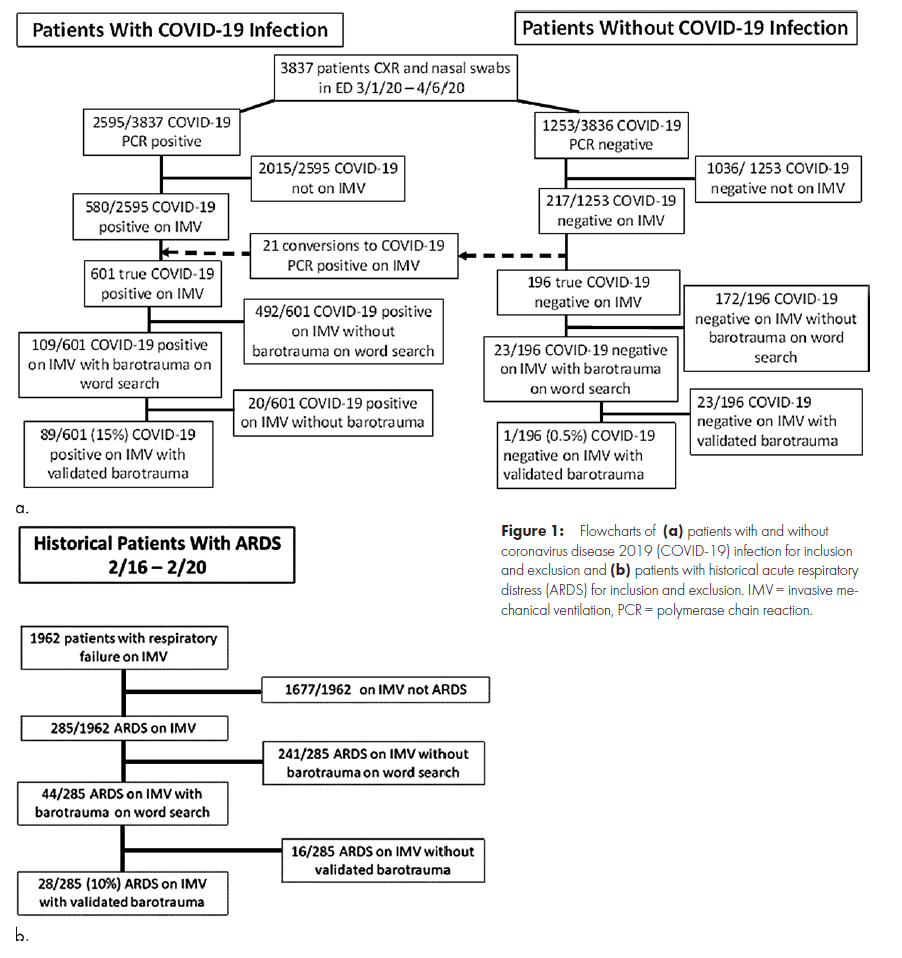 |
| Michelozzi 2020 | North Italy  (10,593,915 residents- include central and south) | Dec 1 2019 - Apr 20, 2020 | 2601 | 5028 | N/A | Confirmed SARS-CoV-2 infection, regardless of pre-existing disease  31 cities in Si SMG corresponding to 53.8% (10,593,915 residents) of urbanized Italian areas, were grouped in into two geographical areas: north (12 cities) and centre and south (19 cities).  total mortality increased abruptly from the beginning of March, reaching a peak at the end of the month, mirrored by the trend in COVID-19 deaths but not in absolute numbers  The proportion of excess death not reported as COVID-19 death increased as the pandemic reached its max,  In the north of Italy, excess mortality in the COVID-19 period (red bars) was greater than COVID- 19 mortality (yellow bars), out of a total of 5028 excess deaths, only 52% were coded as COVID-19. The excess was only marginally explained by the deficit in mortality in the pre-epidemic period  Results by age showed very diverse patterns, with COVID-19 deaths explaining almost all the excess among the youngest groups and only a minor portion among the very old (aged 85+ years). Moreover, a consistent difference by age group was observed: in the youngest groups (15–64 and 65–74 years) COVID-19 deaths account for all the excess, whereas among subjects aged 85+ years only 37% of excess deaths were reported as COVID-19 deaths. It should be noted that in the younger age group the excess is mostly among subjects aged 50 years or more, as reported in other studies  Conversely, the proportion of excess explained by the mortality deficit in the pre-COVID period rapidly declined from 67% in the youngest to 1% to the oldest age group. Results by sex showed a negligible displacement in both men and women (16% of the total excess mortality), with a higher fraction of excess explained by COVID-19 deaths among males (63%).  In this study, age and sex were confirmed as risk factors for COVID-related mortality. The elderly (aged 65+ years) and males show a higher mortality, confirming previous findings  The overlap between total and COVID-19 related mortality was greater among males, suggesting a more severe clinical progression requiring hospitalization, as suggested by the pro-inflammatory pathway activated in old men by the reduced testosterone levels  mortality deficit during the COVID-19 epidemic was exceeded in the northern regions, while in central and southern cities, when taking into account the mortality deficit of previous months, no excess was observed.  Results show that when quantifying the mortality burden related to COVID-19, it is important to consider seasonal dynamics in mortality and possible deficits in previous months |
| Michelozzi 2020  (see above) | Central and South Italy  (10,593,915 residents-include North) | Dec 1, 2019 - Apr 20, 2020 | 522 | 839 | N/A | In central and southern Italy, total excess mortality due to COVID-19 started later on and was more contained, and the gap between total excess mortality and COVID-19 deaths was limited with no clear trend.  Central and southern cities, deficit mortality in the pre-COVID period was greater, and excess mortality during the epidemic was more contained  COVID-19 deaths accounted for 62% of the total excess mortality, with similar differences by age group as in northern cities. The mortality deficit in the pre- COVID period were greater than the excess mortality registered during the outbreak among those aged below 85 years and among females, resulting in proportions of potential displacement greater than 100% in these subgroups  In this study, age and sex were confirmed as risk factors for COVID-related mortality. The elderly (aged 65+ years) and males show a higher mortality, confirming previous findings |
| Miles 2020 | England (single central London hospital) | Up to May 13, 2020 (all cause mortality)  Up to Apr 23, 2020 (Covid-19 + result) | 217 COVID patients compared 160 non-COVID patients | N/A | N/A | Urban teaching hospital aged ≥ 70 tested positive for SARS-CoV-2 by combined throat and high-nasal swab or if there was high clinical suspicion (on the basis of clinical, imaging and laboratory results. control group comprised patients aged ≥ 70 who had been admitted within the same time period who reliably did not have COVID-19.  COVID -19 patients: Age: 80.0 (SD 6.8) (range 70–99) years. The majority of cases were men (*n* = 134, 62%) or of white ethnicity (*n* = 138, 63%).  In univariable models, COVID-19, older age and South Asian ethnicity were associated with higher mortality,  though no measure of socioeconomic position demonstrated any association  For frailty, differences in effect size were evident between cases (HR 1.02, 95% CI 0.93–1.12, *p* = 0.71) and controls (HR 1.99, 95% CI 1.46–2.72, *p* < 0.01).  In multivariable model, these relationships remained consistent: age (HR 1.04, 95% CI  1.01–1.07, *p* < 0.01), South Asian ethnicity (HR 1.13, 95% CI 1.13–3.51, *p* = 0.02)  different associations with frailty according to COVID-19 status was confirmed by demonstrating an interaction term (HR 0.51, 95% CI 0.37–0.71, *p* < 0.01)  The coefficient direction suggest mortality is *proportionally higher* in fitter patients. plotting mutually adjusted survival curves, tertiles of CFS showed distinct trajectories in non-COVID-19 controls, not at all apparent in COVID-19 cases  interaction parameter, differences were most stark in fitter patients (CFS 1–3) but less so in frailer ones (CFS 7–9).  our findings suggest that (1) frailty is not a good discriminator of prognosis in COVID-19 and (2) pathways to mortality may differ in fitter compared with frailer older patients… One might speculate that older people with frailty have pre-existing immunesenescence such that they are unable to mount excess immune responses and may be otherwise by dying from the direct effects of viral infection.  we show that this risk may arise for different reasons depending on pre-morbid frailty |
| Nef 2020 | Germany  (State of Hesse 6 million, 5579 patients from 26 hospital were enrolled) | Mar 23 - Apr 26 2020  Study might require exclusion – data does have mortality but in the context of the pandemic rather than directly on COVID-19 mortalities | 320 | N/A | 5984 vs. 5832 in 2019  Incidence rate = 1.03 p =0.16 | Provide data for all patients who were admitted for cardiac catheterization (exclude: heart failure, arrhythmias, pulmonary embolism): due to chronic coronary syndromes (CCS), ACS (NSTE-ACS, STEMI), and out-of-hospital cardiopulmonary arrest (OHCA). exclude variations for catheterization activities  for other reasons (recent studies, e.g. ISCHEMIA trial; improved primary or secondary prevention) we also analyzed catheter laboratory volumes from January to February 2020 compared with January to February 2019,  COVID-19 death was documented when the patients were tested positive, irrespective of other comorbidities.  22 of 24 public health-authorities in central Germany were aggregated during the pandemic  related lockdown period and compared to the same time period in 2019 (n=5178)  Non-significant increase in all-cause mortality of 2.6% (n=5984 deaths in 2020 time range vs. n=5832 in 2019) [decrease in numbers of death due to other reasons during the lockdown period in 2020 compared with the non-pandemic period in 2019 might be explained by the fact that during the lockdown period there were fewer traffic accidents [24] and less crime [25]. ]  Cardiovascular (comprising cardiac death, pulmonary embolism, and stroke) and significantly by 7.6% (IRR 1.08, 95%-CI 1.01–1.14; *p* = 0.02)  Cardiac mortality increased by 11.8% (IRR 1.12, 95%-CI 1.05–1.19; *p* < 0.001) respectively  The incidence of fatal pulmonary embolism increased by 10.6% (IRR 1.11, 95% CI 0.85–1.44; *p* < 0.46),  Stroke decreased (IRR 0.79, 95% CI 0.66–0.94; *p* = 0.01) and other causes of death (IRR 0.91, 95% CI 0.87–0.95; *p* = 0.001) was lower during the lockdown period in 2020 (in discussions: This finding is may potentially be explained by misdiagnosed or underdiagnosed stroke during lockdown)  During the COVID-19 pandemic, in-hospital mortality in patients admitted for cardiac catherization was higher when compared with 2019 (58/1,801 vs. 55/3,030, *p* = 0.002). Catheterization activities from the high-volume centers  central German State of Hesse, with a population of approximately 6 million, the rate of all-cause mortality increased during the COVID-19 related lockdown compared with the same reference period in 2019. More important, the proportion of cardiovascular deaths increased by 7.6%.  When comparing in-hospital mortality in patients admitted for cardiac catheterization there was an increase in 2020 indicating that these patients were potentially referred too late to the hospital  Causes of death were documented by a physician at the last medical contact. This could mean that an existing COVID-19 diagnosis wrongly failed to state other causes of death. Also: thromboembolic or atherothrombotic events leading to death and listed as the cause of death, e. g. myocardial infarction, could be at least partially related to an existing non-diagnosed COVID-19 infection. … (extensive testing for COVID-19 in central Germany during the lockdown period we assume a negligible number of misdiagnosed patients)  Study guess at reasons patients would have stayed home despite expected higher ACS due to social stressors  The increase hospital mortality -> could have been due to longer referrals  German COVID-19 autopsy study revealed a remarkable rate of deaths (5%) finally defined as non- COVID 19 deaths with virus-independent causes  national programs should be designed to counteract the fact that under such conditions patients at higher cardiovascular risk hesitate to seek cardiac care when symptoms occur |
| Orellana 2020 | Brazil (Manaus) | Epidemiological Week 12-17 | Expressed as ratios – see comments |  | (in males: 1418 deaths in EW14-17 vs. 478 in 2019) ratio 2.97(95%CI 2.67-3.29)  (in females: 878 deaths in EW14-17 vs. 379 in 2019) ratio 2.32(95%CI 2.05-2.61)  Total  2296 deaths in EW14-17 vs. 857 in 2019) ratio 2.68(95%CI 2.48-2.90) | The overall mortality ratio for 2019/2018 is very close to one, independently of EW, while the ratio 2020/2019 only showed a similar pattern (close to one) from EW 12 to 14, with a major increase in the subsequent weeks. In other words, the ratio increased from 1.0 (95%CI: 0.9- 1.3) in EW 12 to 4.6 (95%CI: 3.9-5.3) in EW 17.  overall mortality ratios for 2020/2019 according to age bracket were not statistically signifi­cant in males under 40 years of age or females under 30 years of age, revealing the excess mortality from those age brackets upwards in 2020, especially in males  group of “other causes”, the mortality ratio remained close to one until EW 14, when it increased, reaching a ratio of 3 by EW 17.  Distribution of deaths across age brackets, 69.1% of the deaths occurred in individu­als 60 years or older (95%CI: 66.8-71.4). The ratio of deaths occurring at home or on public byways (43/38) in EW 12 was 1.1 (95%CI: 0.7-1.8). This same ratio (268/33) reached 8.1 (95%CI: 5.7-11.7) in EW 17  EW 15, approximately 15 days after confirmation of the first 30 cases of COVID-19 in Manaus. In EW 17, the anomalous number of deaths coincided with the col­lapse in the public hospital system.  Another key aspect involves gender differences, with a higher risk of mortality among men, cor­roborating findings from other studies  analytical strategy adopted in this study unequivocally reveals the high excess mortality in Manaus and the epidemic’s severity |
| Perkin 2020 | England (1 London teaching hospital) | Mar 12 - April 23  2020 | 243 | 185 | 379 vs. 194 in same 6 weeks in 2019 (p<0.001 – comparison of average weekly rates) | COVID-19-associated deaths where COVID-19 had been identified by a positive swab, and in all cases COVID-19  Group 2 were non-COVID deaths in the same period with no patients in this group having swabbed positive for COVID-19  The total number of deaths in this period in 2020, 379, significantly exceeded the 194 deaths in the same 6 weeks in 2019 (group 3) (comparison of average weekly death rates, p<0.001).  The COVID-19 weekly death rate alone exceeded the 2019 weekly death rate.  Marked reduction in the non-COVID weekly death rate in 2020 and it was significantly lower than the 2019 weekly death rate(p=0.03)  average weekly death rate in the 6 weeks preceding 12th of March 2020, 29 deaths/week, was similar to the 2019 weekly average death rate of 32 deaths/week (p=0.26). Furthermore, while the COVID-19 weekly deaths peaked in the week commencing 2nd of April 2020, the weekly death rate from non-COVID causes continued to diminish across the 6-week period, reaching less than half (14 deaths) of the normal weekly rate by the final week of the period.  Group 1 – COVID19 deaths Group 2 -non-COVID deaths (2020) Group 3 – All deaths (2019)  no significant difference in the sex ratio for non-COVID deaths in 2020 (group 2) or 2019 (group 3)  men were significantly more likely to have died of COVID-19 (67% (162/243), p<0.0001 The excess of men among those dying of COVID-19 was present in every ethnic group (data not shown).  no significant difference in the median age of death between the three groups. Looking at the distribution of ages within each group  median age of death among the 573 cases was: white, 80 years; Asian, 71 years (p<0.001 vs white); Black, 77 years (p=0.05 vs white); Other, 76 years and Not known/not stated, 74 years.  no significant difference within ethnic groups in median age of death comparing those dying of COVID-19 or non-COVID causes in 2020, with the median age of death in 2019  the comparison and p value are COVID-19 vs. 2019, and nonCOVID-19 vs 2019  Compared with deaths in 2019 (46% (90/194) non-white), non-white ethnic groups were significantly over represented among non-COVID deaths in 2020 (58% (79/136), p=0.04), but particularly among COVID-19 deaths (69% (167/243), p<0.001).  COVID-19 deaths in 2020 were more likely to have occurred in those living in the top five deciles of deprivation (44% (108/243) of COVID-19 deaths in 2020 compared with 33% (64/194) of deaths in 2019, p=0.02).  Diabetes significantly more common among patients who died with COVID-19 in 2020 (33% (79/243), p<0.001), compared with deaths in 2019 (16% (32/194)).  Hypertension was more prevalent in both the COVID-19 mortality group (53% (129/243), p=0.02) and the non-COVID deaths in 2020 (55% (75/136), p=0.02), compared with deaths in 2019 (42% (81/194)).  Pre-existing ischaemic heart disease was significantly more prevalent among the non-COVID 2020 deaths compared with the deaths in 2019 (32% (44/136) vs 13% (26/194), p<0.001) (table 1). Multiple comorbidities were significantly more likely in those dying from COVID-19 or non-COVID causes in 2020, compared with deaths in 2019  Compared with 2019, COVID-19 deaths were increased across the ethnic groups: Asian (ORadj=3.62, 95% CI=1.84–7.11, p<0.001); Black (ORadj=2.91, 95% CI= 1.43–5.91, p=0.003) and Other ethnicity (ORadj=3.01, 95% CI=1.61–5.64, p<0.001). Deprivation (IMD>median) was associated with a non-significant increase in likelihood of COVID-19 death, compared with 2019  diabetes mellitus significantly increased the risk of COVID-19 death compared with 2019 (ORadj=1.76, 95% CI=1.05–2.94, p=0.03). There was no significant association with either hypertension or ischaemic heart disease  Male sex remained significantly associated with COVID-19 death (ORadj=2.00, 95% CI=1.32–3.03, p=0.001).  white/non-white resulted in non-white ethnicity being significantly associated with COVID-19 mortality (ORadj=2.43, 95% CI=1.60–3.68, p<0.001).  Compared with deaths in 2019, there was a dose–response relationship between likelihood of death from COVID-19 and increasing number of comorbidities: baseline, no comorbidities (OR=1.0), one comorbidity (ORadj=0.90, 95% CI=0.56–1.44, p=0.67), two comorbidities (ORadj=1.75, 95% CI=0.99–3.10, p=0.06) and three comorbidities (ORadj=3.08, 95% CI=1.00–9.51, p=0.05).  For non-COVID deaths in 2020, all the BAME groups were at increased risk, although with broader CIs given the smaller number of non-COVID deaths: Asian (ORadj=1.59, 95% CI=0.73–3.60, p=0.26), Black (ORadj=2.27, 95% CI=0.98– 5.30, p=0.06) and Other ethnicity (ORadj=3.32, 95% CI=1.66–6.65, p=0.001). Among the individual comorbidities, hypertension (ORadj=1.68, 95% CI=1.03–2.74, p=0.04), and particularly ischaemic heart disease (ORadj=3.41, 95% CI=1.91–6.11, p<0.001), were significantly more likely to be associated with a non-COVID death in 2020 compared with death in 2019. There was no association with sex or deprivation. With ethnicity incorporated as a binary variable, non-white ethnicity was significantly associated with non-COVID death in 2020 (ORadj=1.76, 95% CI=1.09–2.83, p=0.02).  As with COVID-19 deaths, there was a dose-dependent association with increasing numbers of comorbidities leading to a higher likelihood of death from a non-COVID cause in 2020, compared with deaths in 2019: baseline no comorbidities (OR=1.0), one comorbidity (ORadj=1.41, 95% CI=0.84–2.39, p=0.20), two comorbidities (ORadj=2.30, 95% CI=1.19–4.42, p=0.01) and three comorbidities (ORadj=6.46, 95% CI=2.04–20.4, p=0.001).  concern that the drop in non-COVID deaths in the hospital setting may be concealing a rise in such deaths in the community was confirmed in an independent analysis of the Office of National Statistic (ONS) data which demonstrated an absolute and relative increase in non-COVID deaths during the pandemic  no evidence that the elderly were disproportionately represented in the COVID-19 deaths, compared with non COVID- 19 deaths during the same period and also compared with deaths occurring in the same period in the previous year. possible explanation for why we did not see a difference in the age distribution of deaths between groups within the hospital is that our data only relate to deaths in the hospital and not those in the community and a significant proportion of COVID-19 deaths in the UK is occurring in nursing homes or at home  deaths, those living in more deprived areas were over-represented among the COVID-19 deaths, consistent with national data associating poverty with increased likelihood of death from COVID-19 disease. The association was statistically significant in univariate analysis but not in the adjusted analysis  Death from COVID-19 was strongly associated With being man (HR=1.99, 95% CI=1.88–2.10); older age and deprivation (both with a strong gradient); uncontrolled diabetes (HR=2.36 95% CI=2.18–2.56) and severe asthma (HR=1.25 CI=1.08–1.44). Compared with people with ethnicity recorded as white, black people were at higher risk of death, with only partial attenuation in HRs from the fully adjusted model (age sex adjusted HR=2.17, 95% CI=1.84–2.57; fully adjusted HR=1.71, 95% CI=1.44–2.02); with similar findings for Asian people (age-sex adjusted HR=1.95, 95% CI=1.73–2.18; fully adjusted HR=1.62, 95% CI=1.43–1.82). |
| Piccininni 2020 | Italy (Nembro 11 505 as of Jan 1 2020) | Feb 21 - Apr 11  2020 | 85 | N/A (lack of pandemic specific date ranges) | 194 (Jan 1 – Apr 11 2020) vs 112 in 2012, 112 in 2013, 95 in 2014, 119 in 2015, 126 in 2016, 109 in 2017, 128 in 2018, 121 in 2019,  March 2020, monthly all cause mortality  reached a peak of 154.4 per 1000 person years—the  corresponding rate for the same month in 2019 was  14.3 per 1000 person years | study findings show how covid-19 can have a considerable impact on the health of a small community. impact of covid-19 on all cause mortality is especially noticeable when data are analysed from small cities characterised by stable age-sex structures over time and low mobility. This metric is sensitive to small increases in absolute numbers of deaths in small cities.  In March 2020, monthly all cause mortality reached a peak of 154.4 per 1000 person years—the corresponding rate for the same month in 2019 was 14.3 per 1000 person years.  Of the 194 deaths in the first months of 2020, 151 occurred in March alone. Between 21 February and 11 April 2020, a total of 166 deaths were recorded among the residents. (121 in 2019, 128 in 2018)  In April 2020, based on data from the first 11 days, all cause mortality decreased to 23.0 per 1000 person years  More Nembro residents died in March 2020 than in the entire previous year or in any single year since 2012  Since the population of Nembro had been relatively stable across recent years, we conclude that this rapid increase in deaths is attributable to the covid-19 pandemic. Only about half of the deaths observed since the pandemic onset (21 February to 11 April 2020), however, were categorised as confirmed Covid-19 deaths.  does not include date of death, but rather the date the laboratory received the biological sample. This means the number of confirmed covid-19 deaths reported in our study is likely to be slightly higher than the official number in the same period because we included deaths of those who might have died after the 11 of April  For example, a shortage of tests prevented the assessment of covid-19 in people with symptoms and confirmed contacts in Nembro. |
| Richards-Belle 2020 | UK (Scotland excluded) | Feb 1 to Aug 31 2020  vs 2017-2019 (5982 patients) | Critical care (39.1% died n=4240/10834 vs. 20.8% (non COVID -pneumonia n=1203/5782)  Acute hospital (n=4554/10834 42%, mortality for COVID, other viral pneumonias - 1427/5782 24.7%) | NA | NA | Most of COVID-19 deaths occurred before 30 days (88.7%)  Males aged 50–79 years appeared over-represented, when compared with the age/sex distribution for the general population (Fig. 1A), while those of white ethnicity appeared under-represented, when matched on residential location for the general population (Fig. 1B). Critical care patients with COVID-19 were more likely to derive from deprived areas and more likely to be severely obese (BMI ≥ 40) compared with the general population  Mortality increased with increasing age and severity of illness (either decreasing *P*/*F* ratio category or increasing acute physiology score  Compared with critical care patients with COVID-19, median age was similar but a smaller proportion were male (54.3% versus 70.1%) and of non-white ethnicity (11.2% versus 32.6%). Critical care patients with other viral pneumonias (non- COVID-19) were more likely to have needed assistance with daily activities or to have severe conditions prior to hospitalisation than those with COVID-19.  While the generic score (APACHE II) indicated slightly higher acute severity of illness for other viral pneumonias (non- COVID-19), lower *P*/*F* ratios derived from the arterial blood gas with the lowest PaO2 during the first 24 h, indicated greater severity of illness for critical care patients with COVID-19.  In-hospital mortality, whether in the critical care unit or in acute hospital, was lower for critical care patients with other viral pneumonias (non-COVID-19) compared with COVID-19, both overall and when stratified by age/sex, *P*/*F* ratio category, APACHE II acute physiology score and type and combinations of organ support received  clinical diagnosis of COVID-19 in the context of a negative test (less than 1%), where the treating clinical team were convinced that the test was a false negative, were included.  Conventional severity scoring (APACHE II acute physiology score) appeared not to adequately reflect the acute severity of critical care patients with COVID-19. The distribution of critical care patients with COVID- 19 across the more severe *P*/*F* ratio categories appeared to indicate a high degree of acute severity of respiratory disease and much higher than for critical care patients with other viral pneumonias (non-COVID-19), not captured by the APACHE II acute physiology score. |
| Riley 2020 | England (University Hospitals Brimingham NHS foundation Trust) | Apr 1 to Apr 30 2020 vs 2019 | 113 (24.6%) of admission due to COVID-19  N=460 |  | 155 (9.22%) in 2020 vs 79 deaths (3.28%) in 2019 for acute medicine admissions |  |
| Rossen 2020 | USA (CDC National Vital statistics program)  May 30, 2020 N = 331,445,452 (Census.gov) | Jan 26 - Oct 3 2020 vs (same weeks 2015-2019) | 198 081 | 299 028 using average expected number, 224 173 with 95% percentile  Two thirds of excess deaths during the analysis period (66.2%; 198,081) were attributed to COVID-19 and the remaining third to other causes†† | Excess deaths reached their highest points to date during the weeks ending April 11 (40.4% excess) and August 8, 2020 (23.5% excess) | January 26, 2020, through October 3, 2020, an estimated 299,028 more persons than expected have died in the United States. Two thirds of excess deaths during the analysis period (66.2%; 198,081) were attributed to COVID-19 and the remaining third to other causes [number use 2020 vs average expected numbers, if we use 95th percentile of previous value, excess death goes to 224 173). Using the upper bound of the 95% prediction interval for the expected numbers (the upper bound threshold), an estimated 224,173 excess deaths occurred during this period, 85.5% of which were attributed to COVID-19.  Total number of excess deaths (deaths above average levels) from January 26 through October 3 ranged from a low of approximately 841 in the youngest age group (<25 years) to a high of 94,646 among adults aged 75–84 years [Weeks when the observed numbers of deaths were below the average numbers from 2015 to 2019 were excluded from the total numbers of excess deaths above average levels (i.e., negative values were treated as 0 excess deaths]  Average percentage change in deaths over this period compared with previous years was largest for adults aged 25–44 years(26.5%)  Overall, numbers of deaths among persons aged <25 years were 2.0% below average, and among adults aged 45–64, 65–74 years, 75–84, and ≥85 years were 14.4%, 24.1%, 21.5%, and 14.7% above average, respectively.  reason why excess death were below average here vs. above where it mention we had excess death in the youngest group, it was noted that times were excess death were below the average, it was set to 0 instead of a negative number.  White persons, deaths were 11.9% higher when compared to average numbers during 2015–2019. However, some racial and ethnic subgroups experienced disproportionately higher percentage increases in deaths (Figure 3). Specifically, the average percentage increase over this period was largest for Hispanic persons (53.6%). Deaths were 28.9% average for AI/AN persons, 32.9% above average for Black persons, 34.6% above average for those of other or unknown race and 36.6% above average for Asian persons  No p values  Excess deaths reached their highest points to date during the weeks ending April 11 (40.4% excess) and August 8, 2020 (23.5% excess)  The average expected number, as well as the upper bound of the 95% prediction interval (the range of values likely to contain the value of a single new observation), are used as thresholds to determine the number of excess deaths (i.e., observed numbers above each threshold) and percentage excess (excess deaths divided by average expected number of deaths. Estimates described here refer to the number or percentage above the average; estimates above the upper bound threshold have been published elsewhere (*7*).  average numbers of deaths from past years might underestimate the total expected numbers because of population growth or aging, or because of increasing trends in certain causes such as drug overdose mortality |
| Saglietto 2020 | Italy (12 673 821 out of 60 359 546)  21% of pop. | Feb 23 - Mar 21, 2020  vs. (2015-2019) | 4825 | 8750 | 19085 in 2020 vs. (10335)  Overall Mortality rate ratio (MRR) was 1.79 [1.75-1.84] p<0.001  These data are also available for different age brackets | Data included 1084 Italian municipalities, showing at least 10 deaths AND 20% increase in mortality rate vs. mean of last 5 years during Jan 1 - March 21, 2020. Covers 12% of Italian population (12,673,821 out of 60,359,546 inhabitants)  Overall Mortality rate ratio (MRR) was 1.79 [1.75-1.84] p<0.001, excess mortality of 8750, this outweigh Italian Civil protection report of 4825 COVID-19 related deaths across Italy (Feb 23 - March 21, 2020)  Subgroup analysis  No difference in mortality rate in 0-14 age group MRR = 0.68, [0.36-1.28] p=0.21  In those >75, MRR was 1.84 [1.79-1.89] p <0.001  Observing trends, MRR peaked in the last week (March 15 - March 21 2020) with an estimated 2.65 [2.53 - 2.78] p<0.001, subgroup also showed >75 had MRR of 2.75 [2.61-2.90] CI p<0.001  It is uncertain whether the author makes a distinction whether the STEMI case complication is due to COVID-19 infection or due to delayed response. Although the study suggest is it most likely due to behavioural response to lockdown.  Data peaked (March 15 – March 21 2020) – 6753 deaths in 2020, 2546 (2015-2019 average), (MR = 2.65 (95CI% 2.53-2.78) p = <0.001  This study suggest a more reliable method is using all- cause mortality data and comparing them with historical series. |
| Stokes 2020 | USA (1021 counties) | Feb 1 - Sep 23  2020  vs 2013 -2018 | 183 686 Covid-19 deaths | 249 167 (228121 – 270 213 95CI)  65481 (95% 44 435 to 86527) excess death not attributed to COVID-19 |  | First, some direct deaths attributable to Covid-19 may be assigned to other causes of death due to an absence of widespread testing and low rates of diagnoses at the time of death. unfamiliar complications of Covid-19 such as coagulopathy, myocarditis, inflammatory processes, and arrhythmias may have caused confusion and led to attributions of death to other causes, especially early in the pandemic.  Covid -19 death counts do not take into account the indirect consequences of the Covid-19 pandemic on mortality levels: reductions in access to and use of health care services and psychosocial consequences of stay-at-home orders stress, depression, and substance use related to the pandemic could also lead to suicides and overdose deaths. Economic hardship, housing insecurity, and food insecurity may cause indirect deaths, especially among those living with chronic illnesses or who face acute heath emergencies and cannot afford medicines or medical supplies  among other reasons reduce travel deaths, influenza assigned to COVID – 19 instead frailty selection, may die from other disease instead die of COVID-19  alpha – change in mortality independent of COVID-19 mortality, beta1 – past level of all cause mortality, beta2 – extent that past all cause mortality from Covid-19 affect all-cause mortality  estimated value of alpha is 0.572 deaths per 1000 people and beta1 is 0.97 (95% CI, 0.92 to 1.02). Given the observed range of 2013-18 death rates, this combination implies that, when Covid-19 mortality is set at zero, all cause mortality is expected to have risen in all counties between 2013-18 and 2020. Beta 2 is estimated to be 1.36 (95% CI, 1.24 to 1.47). This value suggests that, for every 100 deaths assigned to Covid-19, the number of all-cause deaths rose by 136  26.3% [95% CI, 20.1% to 32.5%] of all excess deaths were not directly assigned to Covid-19 on death certificates  excess deaths were higher than predicted by our model among counties with greater population density and counties that were less rural. For structural factors, excess deaths were higher than predicted by our model among counties with a greater proportion of non-Hispanic Black residents, a lower proportion of non-Hispanic white residents, lower and middle household incomes, greater income inequality, less home ownership, more residents with high housing costs, and more residential segregation  estimates suggest that the majority of excess deaths identified in this study were likely assigned to Alzheimer’s disease and related dementias or various circulatory diseases and diabetes  impact of the Covid-19 pandemic on the Black population is understated when studying data reporting deaths assigned to Covid-19 alone, since counties with higher proportions of Black residents also have more excess deaths not assigned to Covid-19 |
| Strang, Furst 2020 | Sweden (County of Stockholm VAL database)  N= 2,391,990 (https://www.scb.se/en/)you | Mar 1 – May 31 2020 vs (2016-2019) | 1096 (April only) | 2476 (March – May) [(1860+2934+1819) – 6613  (1287+1376+1474)]  -4137  1558 (April) | 1819 vs. 1474 (23%) in March, 2934 vs. 1376 (113%) in April, and 1860 vs.1287 (44%) in May (All Monthly mortality )  590 vs. 531 (11%) in March, 1269 vs. 475(167%) in April, and 625 vs.428 (46%) in May (Nursing home only)  Ex-Nursing home data also available | First, there was a worldwide delay in testing during February and March 2020, meaning that laboratory-confirmed cases only constituted as little as 10–15% of all cases in some countries  any death with a COVID-19 diagnosis according to ICD-10 should be considered as a death from COVID-19  mean age of all the deceased from January to May 2020 was 79.5 years (median 83 years), which was higher than for the deaths during the corresponding months in 2016–2019, 78.8 years (median 82 years), p<0.0001.  In 2020, 49.6% were female, compared with 52.1% for 2016–2019 (chi-square = 18, 1 df), p<0.0001  Whereas January and February were similar to the calculated means, the proportions of deaths were significantly higher for March, April, and May (23%, 113%, and 44%, respectively), p<0.0001  Only patients over 80 years of age had excess deaths in March, whereas all the studied age groups were affected in April. In May, excess deaths were mainly attributed to patients aged 70–79 years and to those aged 80 years or more (40-59,60-69,70-79,80+)  Proportion of patients dying in nursing homes as a fraction of all deaths in 2020 was 32% in March, 43% in April, and 34% in May. When specifically studying the percentage of excess deaths in nursing homes, compared with deaths in 2016–2019, the proportions were found to be significantly higher: 11% in March, 167% in April, and 46% in May  excess deaths (all causes) for March–May, for those dying in other places than nursing homes. In March, the excess deaths (%) were higher for other places of deaths than nursing homes, 30% versus 11%  proportions of deaths with a registered COVID-19 diagnosis compared with all deaths were 10% for March, 37% for April, and 32% for May.  COVID-19-related deaths were also calculated in relation to every 1000 inhabitants, stratified both for Mosaic groups and for age groups  In April 1096 confirmed COVID-19 deaths and a total of 2934 deaths. When removing the number of those who died from COVID-19, the remaining number is 1838 deaths in April, which is significantly higher than for the reference years (95% CI 1300–1452).  Changes in place of care for nursing home residents were mapped for the last two weeks of life, for the months March–May. In a first comparison, we studied trends for 2016–2019 compared with 2020. For the period 2016 2019, 28.3% (95% CI 26.7%–30.0%) had at least one change as regards place of care, compared with 15.2% for the corresponding period in 2020 (chi-square = 162, 1 df; p<0.0001)  Residents dying from causes other than COVID-19 had more changes of place in May than in April (Table 7). Whereas 5% were referred acutely during the last two weeks of life in April, the corresponding figure had increased to 13% in May (chi-square = 24.6, 1 df; p<0.0001).  proportion of nursing home residents who eventually died either in an acute hospital or in a geriatric hospital ward, the proportion for residents dying from COVID-19 during March–May was 19%, and for residents dying from other causes 5% (chi-square = 109, 1 df; p<0.0001)  [it is obvious that the total number of changes was lower in 2020, implying that there was a general reluctance to admit dying residents to acute hospitals although 24% of those dying from COVID-19 and 12% of the others were acutely admitted to hospitals during their last two weeks of life, only 19% and 5% of these patients actually died in hospitals, implying that some of them were sent back to the nursing homes]  Discussion:  peak month (April) was most pronounced in these groups, with 86% of the COVID-19-related deaths in persons over 70 years of age  In many studies, socio-economic status and belonging to a minority are strongly intercorrelated, but the risk of contracting COVID-19 and dying from the disease is not explained merely by socio-economic status or comorbidities. As shown by Lassale et al. in their study, black individuals had an increased risk of COVID-19 infection compared with white individuals, even when adjusting for age, sex, and other potential explanatory factors which included neighbourhood deprivation, household crowding, smoking, body size, inflammation, glycated haemoglobin, and mental illness (22). Similar data have been published by Williamson et al. (23). |
| Vestergaard 2020 | Europe (24 European countries)  Austria, Belgium, Denmark, England (UK),  Estonia, Finland, France, Germany (Berlin and Hesse),  Greece, Hungary, Ireland, Italy (19 cities), Luxembourg,  Malta, the Netherlands, Northern Ireland (UK), Norway,  Portugal, Scotland (UK), Spain, Sweden, Switzerland  and Wales (UK).  EuroMOMO | Week 1 – Week 18  Jan 1 –May 3  2020 vs (2016-2019) | NR | At the peak level of  mortality, in week 14, an excess of 35,802 deaths across all ages was estimated, of which 32,815 (92%) were persons aged ≥ 65 years | Cumulative excess mortality from week 1 to week 18/2020 reached a total of 185,287 deaths  (all ages), including 24,438 (13%) in persons aged  65–74 years, 55,226 (30%) in persons aged 75–84 years,  and 88,598 (48%) in persons aged ≥ 85 years (wk1-wk18)  younger age groups reached 14,339 (8%) in 45–64 year-old persons and 1,843 (1%) in 15–44 year-old persons (wk1-wk18)  vs. Baseline:  The cumulative excess deaths (all ages) by week 18 reached 55,441 in 2019, 110,483 deaths in 2018, 83,009 deaths in 2017 and 29,849 deaths in 2016 | Official national statistics on COVID-19 cases and deaths among European countries are heterogeneous, partly due to the differences in applied testing strategies and access to testing, and use of different reporting modalities. In this situation, numbers of excess all-cause deaths can provide a more complete and timely proxy measure of the mortality burden of COVID- 19 in the population,  Excess mortality particularly affected ≥ 65 year olds (91% of all excess deaths), but also 45–64 (8%) and 15–44 year olds (1%). No excess mortality was observed in 0–14 year olds  Mortality estimate showed an increasing trend during the first weeks of March 2020, and an excess mortality level higher than four z scores above the baseline (defined as ‘substantial excess’) in week 11 (9–15 March)/2020  Mortality increased steeply in the next 3 weeks and peaked in all countries during week 14 (30 March–5 April)/2020, when a total of 88,581 deaths (all ages) was reached, translating into a z-score of 58. By week 15 (6–12 April)/2020 the mortality started a rapid decline, affecting all age groups except the 0–14 years where no excess mortality had been observed; however, by week 18/2020 a substantial mortality for all ages combined, of around 60,000 deaths, was still seen, corresponding to a z-score of 16 |
| Vieira 2020 | Portugal (Portuguese Death Certificate Information System (SICO-eVM)  Worldbank N= 10,305,564 | Mar 16 – Apr 14 2020 vs (Jan 1 - Apr 14 (last 10 yrs), age and cause (last 6 years) | 599 deaths (18051 cases) | 1255 deaths, 14% more than expected (previous 10 year daily average)  1214 (if using ARIMA modelling) | 13.7% vs. baseline (previous 10 years) | 49% of the estimated excess deaths were registered as due to COVID-19, the other 51% registered as other natural causes.  March 2020, 187 people died of COVID-19, or 2.3% of the 8521 confirmed patient cases, a cumulative incidence of around 80 cases per 100,000 inhabitants  10 year data for: March and April showed declining mortality in relation to January and February. In 2020, there is an inversion of this negative trend as of March 11, with an EM above the average of the previous 10 years,  75 years or more. Between March 1 and April 14, 2020, there was an EM of 1030 deaths in people aged 75+, compared to the daily averages of deaths in this age group, in the last 6 years  In the same period, only 67 deaths were estimated above the histor­ical average in the age group between 65 and 74 years old and EM was not identified in any other age group  Between March 16 and April 14, there was an EM of 1281 deaths from natural causes, based on deaths registered in the last 6 years  Between March 16 and April 14, there was a 15.2% increase in deaths from natural causes and a 57% reduction in deaths from external causes compared to the averages observed in the previous 6 years  excess mortality estimates are conservative, since expected deaths are influenced by the previous years and the present year reg­istered lower mortality than expected in February and early March  Our study shows a decline in excess mortality 2 weeks after it imple­mented stringent confinement measures, with which the population complied |
| Woolf 2020 | USA (excl. Connecticut, North Carolina) | Mar 1 - Apr 25  2020 | 56 246 (65% of excess deaths) | 87 001 (95%CI 86 578 – 87423)  73524 - if modelling using epidemiologic years | 505 059 deaths vs. expected deaths 419058 | 5 states with the most COVID-19 deaths experienced large proportional increases in deaths due to non respiratory underlying causes, including diabetes (96%), heart diseases (89%), Alzheimer disease (64%), and cerebrovascular diseases (35%)  Expected deaths (and 95% CIs) for these same weeks were estimated by fitting a hierarchical Poisson regression model to the weekly death counts for the period of December 29, 2013, through February 29, 2020 (assembled from final data for 2014-20181 and provisional data for January 1, 2019, through February 29, 20202). The model with the optimal fit (Supplement) used a combination of harmonic functions to capture seasonality and adjusted for annual trends with a categorical year effect. The model allowed season and time trends to vary by state |
