# Supplementary material for: Coronavirus disease 2019 (COVID-19) excess mortality outcomes associated with pandemic effects study (COPES): A systematic review and meta-analysis
Source: Front Med (Lausanne). 2022 Dec 16;9:999225. doi: 10.3389/fmed.2022.999225 (PMC9800609; doi:10.3389/fmed.2022.999225)
Supplement: Supplementary file 10 [file Table_4.docx]

**Supplemental Table 4A: Risk of Bias Assessment for Observational Cohort Studies – Newcastle-Ottawa Score**

| Studies: | Selection  (max 4 stars) | Comparability  (max 2 stars) | Outcome  (max 3 stars) | Total  (max 9 stars) | Study Quality  Grade |
| --- | --- | --- | --- | --- | --- |
| Alicandro 2020 | 4 | 0 | 3 | 7 | Poor |
| Bilinski 2020 | 4 | 0 | 2 | 6 | Poor |
| Birkmeyer 2020 | 4 | 1 | 1 | 6 | Poor |
| Blangiardo 2020 | 4 | 1 | 2 | 7 | Good |
| Bustos Sierra 2020 | 4 | 0 | 1 | 5 | Poor |
| Cates 2020 | 4 | 1 | 1 | 6 | Poor |
| Cevallos-Valdiviezo 2020 | 4 | 0 | 1 | 5 | Poor |
| Conti 2020 | 4 | 0 | 1 | 5 | Poor |
| Cusack 2020 | 4 | 0 | 3 | 7 | Poor |
| Friedman 2020 | 4 | 0 | 3 | 7 | Poor |
| Habonimana 2020 | 4 | 2 | 3 | 9 | Good |
| Jacobson 2020 | 4 | 0 | 3 | 7 | Poor |
| Lerner 2020 | 4 | 0 | 3 | 7 | Poor |
| Little 2020 | 4 | 0 | 1 | 5 | Poor |
| Mannucci 2020 | 4 | 1 | 3 | 8 | Poor |
| McGuinness 2020 | 4 | 1 | 1 | 6 | Poor |
| Michelozzi 2020 | 4 | 0 | 1 | 5 | Poor |
| Miles 2020 | 4 | 0 | 1 | 5 | Poor |
| Nef 2020 | 4 | 0 | 1 | 5 | Poor |
| Orellana 2020 | 4 | 0 | 1 | 5 | Poor |
| Piccininni 2020 | 4 | 0 | 1 | 5 | Poor |
| Richards-Belle 2020 | 3 | 0 | 1 | 4 | Poor |
| Riley 2020 | 4 | 0 | 1 | 5 | Poor |
| Rossen 2020 | 4 | 0 | 3 | 7 | Poor |
| Saglietto 2020 | 4 | 0 | 1 | 5 | Poor |
| Stokes 2020 | 4 | 0 | 1 | 5 | Poor |
| Strang 2020 | 4 | 0 | 1 | 5 | Poor |
| Vestergaard 2020 | 4 | 0 | 1 | 5 | Poor |
| Vieira 2020 | 4 | 1 | 1 | 6 | Poor |
| Woolf 2020 | 4 | 1 | 1 | 6 | Poor |

Non-randomized observational trials were assessed for ROB using the Newcastle-Ottawa Scale (NOS), examining the following domains: selection (max score of 4), comparability (max score of 2) and exposure (max score of 3) for cohort studies (Wells 2019). Quality of the studies were based on either good (3-4 stars in selection domain and 1-2 stars in comparability domain and 2-3 stars in outcome/exposure domain), fair (2 stars in selection domain and 1-2 stars in comparability domain and 2-3 stars in outcome/exposure domain) or poor (0-1 star in selection domain or 0 stars in comparability domain or 0-1 stars in outcome/exposure domain) quality (Wells 2019).

*Selection*

1. Representativeness of intervention cohort:
   1. Truly representative of average, treated probiotic patient in hospital*
   2. Somewhat representative of average, treated probiotic patient in hospital*
   3. Only selected group of patients
   4. No description of derivation cohort
2. Selection of non-intervention cohort:
   1. Drawn from same community as intervention/exposed cohort*
   2. Drawn from different source
   3. No description of the derivation of the non-exposed cohort
3. Ascertainment of intervention:
   1. Health record*
   2. Structured interview*
   3. Written self-report
   4. No description
4. Demonstration that outcome of interest was not present the start of the study:
   1. Yes*
   2. No

*Comparability*

1. Comparability of cohorts on the basis of the design or analysis:
   1. Study controls for baseline demographics (e.g. age)*
   2. Study controls for an additional factors (e.g. illness severity)*

*Outcome*

1. Assessment of outcome:
   1. Independent blind assessment*
   2. Record linkage*
   3. Self report
   4. No description
2. Was follow-up long enough for outcomes:
   1. Yes (median duration of follow-up 4 weeks)*
   2. No
3. Adequacy of follow-up cohort:
   1. Complete follow-up*
   2. Minimal loss to follow-up (<20%)*
   3. Follow-up rate <80% and no description of losses to follow-up
   4. No statement

**Supplemental Table 4B: Risk of Bias Assessment for Observational Case-Control Studies – Newcastle-Ottawa Score**

| Studies: | Selection  (max 4 stars) | Comparability  (max 2 stars) | Outcome  (max 3 stars) | Total  (max 9 stars) | Study Quality  Grade |
| --- | --- | --- | --- | --- | --- |
| Perkins 2020 | 4 | 2 | 2 | 8 | Good |

Non-randomized observational trials were assessed for ROB using the Newcastle-Ottawa Scale (NOS), examining the following domains: selection (max score of 4), comparability (max score of 2) and exposure (max score of 3) for cohort studies (Wells 2019). Quality of the studies were based on either good (3-4 stars in selection domain and 1-2 stars in comparability domain and 2-3 stars in outcome/exposure domain), fair (2 stars in selection domain and 1-2 stars in comparability domain and 2-3 stars in outcome/exposure domain) or poor (0-1 star in selection domain or 0 stars in comparability domain or 0-1 stars in outcome/exposure domain) quality (Wells 2019).

*Selection*

1. Is case definition adequate?
   1. Yes, with independent validation*
   2. Yes, e.g. record linkage or based on self reports*
   3. No description
2. Representativeness of cases:
   1. Consecutive or obvious representativeness of cases*
   2. Potential for selection biases or not stated
3. Selection of controls
   1. Community controls*
   2. Hospital controls
   3. No description
4. Definition of controls:
   1. No history of disease (endpoint)*
   2. No description of source

*Comparability*

1. Comparability of cases and controls on the basis of the design or analysis:
   1. Study controls for age * (select the most important factor)
   2. Study controls for any additional factors* (This criteria could be modified to indicate specific control for a second important factor)

*Outcome*

1. Ascertainment of outcome:
   1. Secure record (e.g. surgical records)*
   2. Structured interview where blind to case/control status*
   3. Interview where not blind to case/control status
   4. Written self-report or medical record only
   5. No description
2. Same method for ascertainment for cases and controls:
   1. Yes*
   2. No
3. Non-response rate:
   1. Same rate for both groups*
   2. Non-respondents described
   3. Rate different and no designation
