# Supplementary material for: Coronavirus disease 2019 (COVID-19) excess mortality outcomes associated with pandemic effects study (COPES): A systematic review and meta-analysis
Source: Front Med (Lausanne). 2022 Dec 16;9:999225. doi: 10.3389/fmed.2022.999225 (PMC9800609; doi:10.3389/fmed.2022.999225)
Supplement: Supplementary file 11 [file Table_5.docx]

**Supplemental Table 5:** Grading of Recommendations Assessment, Development and Evaluation (GRADE) of COPES Outcomes: COVID-19 mortality, all-cause excess mortality, non-COVID-19 mortality

| **Certainty assessment** | | | | | | | **Impact** | **Certainty** | **Importance** |
| --- | --- | --- | --- | --- | --- | --- | --- | --- | --- |
| **№ of studies** | **Study design (sources)** | **Risk of bias** | **Inconsistency** | **Indirectness** | **Imprecision** | **Other considerations ^a^** |  |  |  |
| COVID-19 Mortality | | | | | | | | | |
| 28 | Observational studies  (27 cohort, 1 case-control)  N=430,940,442 | very serious ^b^ | not serious ^c^ | not serious ^d^ | not serious ^e^ | none ^f^ | - The change in mortality outcome was reported in 28 studies of which 4 studies reported a statistically significant increase in mortality rate in COVID-19 positive patients, while 2 found no statistically significant increase in mortality rate. - RoB was rated as “very serious” – given the high proportion of poor NOS vs. good NOS scores - There was not serious inconsistency in this SR. 2 studies did not find statistically significant mortality difference. In one, COVID-19 related symptoms were used as a criterion and no information related to tested was presented. The other had age confounders which likely impacted mortality results - Indirectness was rated not serious because we are using our metric of interest (mortality) directly in our assessment. - Imprecision was assessed using a subset with population-based data. Given our large data set and small confidence interval, this was rated non serious. - Given all observational studies start at a “low certainty rating”, plus downgrades for RoB, we would consider the certainty in the evidence to be “very low” quality for mortality | ⨁◯◯◯ Very low quality | CRITICAL |
| All cause mortality | | | | | | | | | |
| 27 | Observational studies  (26 cohort, 1 case-control  N=433,196,345 | very serious ^b^ | not serious ^c^ | not serious ^d^ | not serious ^e^ | none ^f^ | - The change in mortality outcome was reported in 27 studies of which 17 found significant all cause mortality increase, 9 found increases in all cause mortality, and 1 found no difference - RoB was rated as “very serious” – given the high proportion of poor NOS vs. good NOS scores - There is not serious inconsistency in this literature (most studies found significant increases (63% of had statistically significant mortality difference, 33% found increases while 3.7% found no change). - Indirectness was rated not serious because we are using mortality data directly in our assessment. - Imprecision was rated as not serious, given that all the studies used in the meta-analysis did not have its 95% CIs cross 1 - Given all observational studies start at a “low certainty rating”, plus downgrades for RoB, we would consider the certainty in the evidence to be “very low” quality for all cause mortality | ⨁◯◯◯ Very low quality | CRITICAL |
| Non-COVID-19 mortality | | | | | | | | | |
| 16 | Observational studies  (15 cohort, 1 case-control)  N=430,940,442 | very serious ^b^ | not serious ^c^ | not serious ^d^ | not serious ^e^ | none ^f^ | - The change in non-COVID-19 mortality outcome was reported in 16 studies of which 10 studies found increases in non-COVID-19 mortality, 6 studies found significant increases in non-COVID mortality - RoB was rated as “very serious” – given the high proportion of poor NOS vs. good NOS scores - There is not serious inconsistency in this literature (94% of studies found some increase in non-COVID-19 mortality). - Indirectness was rated not serious because we are using mortality data directly in our assessment. - Imprecision was rated as not serious, given none of the studies used in the meta-analysis cross 1, while all the p-values or 95% CIs in the meta-analysis do show significance differences - Given all observational studies start at a “low certainty rating”, plus downgrades for RoB, we would consider the certainty in the evidence to be “very low” quality for non-COVID-19 mortality | ⨁◯◯◯ Very low quality | CRITICAL |

**CI:** confidence interval, **GRADE:** Grading of Recommendations Assessment, Development and Evaluation, **NOS:** Newcastle-Ottawa Scale, **RoB:** risk of bias, **SR:** systematic review

a. Other considerations: e.g., publication bias, large magnitude of effect, dose-response gradient, all plausible confounding would reduce the demonstrated effect or increase the effect if no effect was observed

b. “Very serious” rating based on poor RoB in 90.3%, and only good in 9.7% of all studies (n = 31)

c. “Not serious” rating based on overall inconsistency (specifically there are minimal discrepancies for differences in all outcomes: Covid-19 mortality (66.7% had vs. 33.3% not), all cause mortality (96.3% had increase vs. 3.7% not) and non-covid mortality (93.8% had increase vs. 6.2% not)

d. “Not serious” rating for indirectness, given all studies measured directly at the 2 *a priori* outcomes (Covid-19 mortality, all cause mortality and non-Covid-19 mortality)

e. “Not serious rating” for imprecision, as the confidence intervals from studies used in the meta-analysis does not cross 1

f. There is unlikely to be any significant other considerations. Publication bias is unlikely to be present, given the extensive search during this SR. Furthermore, there is also no consistent large magnitude of effect, dose-response gradient
