# Supplementary material for: Coronavirus disease 2019 (COVID-19) excess mortality outcomes associated with pandemic effects study (COPES): A systematic review and meta-analysis
Source: Front Med (Lausanne). 2022 Dec 16;9:999225. doi: 10.3389/fmed.2022.999225 (PMC9800609; doi:10.3389/fmed.2022.999225)
Supplement: Supplementary file 12 [file Table_6.docx]

**Supplemental Table 6:** Demographic based mortality data summary

| Variable | Study | Result |
| --- | --- | --- |
| Age | Rossen 2020 | - All cause mortality was 2% below baseline in those below 25 while adults aged 45–64, 65–74 years, 75–84, and ≥85 years were 14.4%, 24.1%, 21.5%, and 14.7% above average, respectively |
|  | Vestergaard 2020 | - Mortality was highest among individuals aged 65 years and older, but some countries also observed marked excess deaths among those aged 45–64 years, and some countries (in particular England and Spain) even noted excess mortality in the age group 15–44 years, also reflected in the overall pooled estimates. No excess mortality was observed in children aged 0–14 years. |
|  | Orellana 2020 | - Excess overall mortality was observed with increasing age, es­pecially in individuals 60 years or older, who accounted for 69.1% (95%CI: 66.8-71.4) of the deaths. - no significant increase in mortality in males under 40 or females under 30 years of age |
|  | Jacobson 2020 | - Showed a significant decrease (45.3 weekly non-COVID death in 2019 vs 37.5 in 2020) for female 5-14 suggesting COVID-19 was protective in this population (p<0.001) |
|  | Conti 2020 | - In the age-class 70–79, the number of all cause cumulative deaths increased by 5.05-fold (95% CS 4.83–5.28), compared to 2.18-fold (95% CS 1.96–2.44) among the youngest (less than 60) |
|  | Saglietto 2020 | - Mortality rate ratio (MRR) peaked in with an estimated 2.65 [2.53 - 2.78] p<0.001, with subgroup analysis showing >75 had MRR of 2.75 [2.61-2.90] CI p<0.001 while those 15-64 had a MRR of 1.65 ([1.40-1.95] CI p<0.001 |
|  | Strang 2020 | - Excess death in March only in those over 80, while all age range studied (40-59,60-69,70-79,80+) had significant excess death in April and only those in 70-79 and over 80 had excess death in May. |
|  | Michelozzi 2020 | - Excess deaths were found but the percent that can be accounted by COVID-19 varied by age, COVID-19 deaths explained 98% of all excess death among the youngest (15-64) group but only 37% of those over age 85. The author notes that the younger age group, the excess is mostly among people aged 50 years or more |
|  | Vestergaard 2020 | - Identified predominately populations over 65 made up of 91% of all excess death, those 15-44 made up 8% and 15-44 year old made up 1%. |
|  | McGuinness 2020 | - Association of mortality in patients with COVID-19 infection with greater age (odds ratio= 1.05; *P* , .001) |
|  | Miles 2020 | - Multivariable model for age: hazard ratio of 1.04 CI 95% CI 1.01–1.07 p<0.01 |
|  | Richards-Belle 2020 | - Found mortality increased with age (represented graphically) |
|  | Strang 2020 | - Indirectly indicated increased mortality among older people, finding the mean age of all deceased increased from 78.8 to 79.5 with a p value of less than 0.0001 |
|  | Perkin 2020 | - Found no evidence that the elderly were disproportionately represented in the COVID-19 deaths, compared with non COVID- 19 deaths during the same period and also compared deaths occurring in the same period in the previous year. Perkin outlined a possible explanation for this lack of difference by attributed the data only included deaths in the hospital and not those in the community (nursing homes or at home) which made up a significant proportion of COVID-19 deaths in the UK |
| Sex | Strang 2020 | - 49.6% of all deaths were female in 2020 versus baseline of 52.1 (P<0.0001) |
|  | Perkin 2020 | - Significant relationship where 67% of COVID-19 mortality was male versus non-COVID population during the same time period was 53% p<0.001 - Adjusted odds ratio of 2.00 death (ORadj=2.00, 95% CI=1.32–3.03, p=0.001) for male sex |
|  | Orellana 2020 | - Overall mortality ratios for 2020/2019 according to age bracket were not statistically signifi­cant in males under 40 years of age or females under 30 years of age, revealing the excess mortality from those age brackets upwards in 2020, especially in males |
|  | Michelozzi 2020 | - Results by sex showed a negligible displacement in both men and women (16% of the total excess mortality), with a higher - Fraction of excess explained by COVID-19 deaths among males (63%). |
|  | Perkin 2020 | - Death from COVID-19 was strongly associated male (HR=1.99, 95% CI=1.88 – 2.10) |
|  | Jacobson 2020 | - Men aged (15-24, 25-34, 35-44 and 45-54 years) show a significant increase in expected non-COVID-19 weekly while deaths for males aged 5-14, 75-84 and >85 years and for females aged 15-24, 45-54, 55-64, 65-74, 75-84 and >85 years the increased deaths rate could be explained by COVID-19 alone. - For women, two age cohorts (25-34 and 35-44 years) show a statistically significant (P-value < 0.001) increase in expected 2020 non-COVID-19 weekly deaths compared with the 2019 weekly deaths |
| Racial minorities | Rossen 2020 | - Mortality had increased across all races versus previous years but found disproportional increases. White persons had the lowest increase at 11.9% while highest increase was in Hispanics at 53.6%. In between, 28.9%, 32.9% and 34.6%, 36.6 above average mortality was found for non-Hispanic American Indian or Alaska Native, Black persons, other or unknown race and Asian persons respectively. |
|  | McGuinness 2020 | - Risk of death in the context off acute respiratory distress syndrome finding Hispanic ethnicity had an odds ratio of 1.73, p = 0.05 |
|  | Miles 2020 | - Hazard ratio of 1.13 p=0.02 for South Asians for multivariable model for COVID-19 mortality |
|  | Perkin 2020 | - Non-white associated with an adjusted odds ratio of 2.43 p<0.001), finding non-white ethnic groups were significantly over represented among non-COVID deaths in 2020 (58% vs 46% in 2019, p=0.04) and particularly among COVID-19 death (69% p<0.001). - Increased mortality across the ethnic groups [Adjusted odds ratios (ORadj)]: Asian (ORadj=3.62, 95% CI=1.84–7.11, p<0.001); Black (ORadj=2.91, 95% CI= 1.43–5.91, p=0.003) and Other ethnicity (ORadj=3.01, 95% CI=1.61–5.64, p<0.001) - For non-COVID deaths in 2020, all the BAME groups were at increased risk, although with broader CIs given the smaller number of non-COVID deaths: Asian (ORadj=1.59, 95% CI=0.73–3.60, p=0.26), Black (ORadj=2.27, 95% CI=0.98– 5.30, p=0.06) and Other ethnicity (ORadj=3.32, 95% CI=1.66–6.65, p=0.001). - With ethnicity incorporated as a binary variable, non-white ethnicity was significantly associated with non COVID death in 2020 (ORadj=1.76, 95% CI=1.09–2.83, p=0.02). |
|  | Cates 2020 | - The per­centage of COVID-19 patients who died while hospitalized (21.0%) was more than five times that of influenza patients (3.8%), and the duration of hospitalization was almost three times longer for COVID-19 patients. Among patients with COVID-19, the risk for respiratory, neurologic, and renal complications, and sepsis was higher among non-Hispanic Black or African American (Black) patients, patients of other races, and Hispanic or Latino (Hispanic) patients compared with those in non-Hispanic White (White) patients, even after adjusting for age and underlying medical conditions. |
| Socioeconomic status (SES) | Perkin 2020 | - Utilized a deciles of deprivation scale finding COVID-19 deaths were more likely to be found in the top five deciles were more likely 44% (108/243) of COVID-19 deaths in 2020 compared with 33% (64/194) of deaths in 2019, p=0.02) while non-COVID deaths had similar distributions - Attempted to distinguish confounding factors between lower SES and non-white individuals finding an association with COVID-19 death on univariate analysis but not in the adjusted analysis indicating a confounder between lower SES and non-white individuals |
|  | Strang 2020 | - Stratified SES into mosaic group between 1 and 3 and found significant differences with more deaths in the least affluent area |
|  | Stokes 2020 | - Excess deaths were higher than predicted among USA counties with a greater proportion of non-Hispanic Black residents, a lower proportion of non-Hispanic white residents, lower and middle household incomes, greater income inequality, less home ownership, more residents with high housing costs, and more residential segregation - Estimates suggest that the majority of excess deaths identified in this study were likely assigned to Alzheimer’s disease and related dementias or various circulatory diseases and diabetes. It is possible that a substantial fraction of the deaths of individuals with pre-existing chronic conditions who acquire Covid-19 and die as a result are ascribed to the pre-existing condition. These may constitute many of the excess deaths not attributed to Covid-19. |
|  | Birkmeyer 2020 | - Stratified analysis of in-hospital mortality (with coefficients reported in the appendix and adjusting for age, sex, and diagnosis),17 suggested that much of the increase in in-hospital mortality for non-COVID admissions during April occurred for patients living in majority-Black, majority-Hispanic, or high poverty ZIP codes. In April, mortality in this subgroup was 0.5 percent higher than for those not in the subgroup (p= 0.03). By the end of May, that disparity in mortality had disappeared. |
|  | Friedman 2020 | - Although respiratory cases were strikingly concentrated in the high- and highest-SES quintiles, the highest out-of-hospital mortality rates were observed in low-SES areas. There is a notable difference between respiratory cases and deaths, which may suggest that the profile of individuals who have the economic or social capital to seek care early for respiratory symptoms in Tijuana differs from those who do not interact with the medical system until after their death |
|  | Miles 2020 | - Index of multiple deprivation did not find association between increased mortality for COVID-19 |
| Comorbidities | Perkin 2020 | - Compared with deaths in 2019, there was a dose–response relationship between likelihood of death from COVID-19 and increasing number of comorbidities: baseline, no comorbidities (OR=1.0), one comorbidity (ORadj=0.90, 95% CI=0.56–1.44, p=0.67), two comorbidities (ORadj=1.75, 95% CI=0.99–3.10, p=0.06) and three comorbidities (ORadj=3.08, 95% CI=1.00–9.51, p=0.05) - Diabetes significantly more common among patients who died with COVID-19 in 2020 (33% (79/243), p<0.001), compared with deaths in 2019 (16% (32/194) - Hazard ratio for uncontrolled diabetes (HR=2.36 95% CI=2.18–2.56) and severe asthma (HR=1.25 CI=1.08–1.44) - Among the individual comorbidities, hypertension (ORadj=1.68, 95% CI=1.03–2.74, p=0.04), and particularly ischaemic heart disease (ORadj=3.41, 95% CI=1.91–6.11, p<0.001), were significantly more likely to be associated with a non-COVID death in 2020 compared with death in 2019 |
|  | Cusack 2020 | - 139 death with Covid-19, 137 (99%) had underlying conditions: 80 (58%) cardiovascular (including hypertension); 78 (57%) dementia; 30 (22%) respiratory; 19 (43%) neurological; 17 (12%) oncological; 15 (11%) diabetes; and 9 (7%) renal |
|  | McGuinness 2020 | - Focused on barotrauma but did not find survival differences between patients with COVID-19 with or without barotrauma. The study attribute this to younger age in the barotrauma cohort that may have negated this factor. However, when comparing historical ARDS, COVID-19–negative, and COVID-19–positive groups (*P <*.001), with patients in the historical ARDS group showing the longest survival - multivariate analysis did find mortality in with patients sustaining barotrauma had an odds ratio of 2.2, p=0.03 |
|  | Miles 2020 | - Examined those over 70 admitted to an urban hospital and offers a contradictory view, its model suggest that mortality was proportionally higher in fitter patients. Miles suggest that frailty is not a good discriminator of prognosis and speculate that older people with frailty have pre-existing immuesenescence is unable to mount an excessive immune response and may be dying for the direct effects of infection. They estimate the hazard ratio in this population to be 213, p<0.01. |
|  | Strang 2020 | - Proportion of patients dying in nursing homes as a fraction of all deaths in 2020 was 32% in March, 43% in April, and 34% in May. When specifically studying the percentage of excess deaths in nursing homes, compared with deaths in 2016–2019, the proportions were found to be significantly higher: 11% in March, 167% in April, and 46% in May. |
